# Comparative population genomics reveals convergent and divergent selection in the apricot–peach–plum–mei complex

**DOI:** 10.1093/hr/uhae109

**Published:** 2024-04-16

**Authors:** Xuanwen Yang, Ying Su, Siyang Huang, Qiandong Hou, Pengcheng Wei, Yani Hao, Jiaqi Huang, Hua Xiao, Zhiyao Ma, Xiaodong Xu, Xu Wang, Shuo Cao, Xuejing Cao, Mengyan Zhang, Xiaopeng Wen, Yuhua Ma, Yanling Peng, Yongfeng Zhou, Ke Cao, Guang Qiao

**Affiliations:** Key Laboratory of Plant Resource Conservation and Germplasm Innovation in Mountainous Region (Ministry of Education), Institute of Agro-bioengineering/College of Life Sciences, Guizhou University, Guiyang 550025, China; National Key Laboratory for Germplasm Innovation & Utilization of Horticultural Crops, Zhengzhou Fruit Research Institute, Chinese Academy of Agricultural Science, Zhengzhou 450009, China; National Key Laboratory of Tropical Crop Breeding, Shenzhen Branch, Guangdong Laboratory of Lingnan Modern Agriculture, Key Laboratory of Synthetic Biology, Ministry of Agriculture and Rural Affairs, Agricultural Genomics Institute at Shenzhen, Chinese Academy of Agricultural Sciences, Shenzhen 518120, China; College of Horticulture and Forestry Sciences, Huazhong Agricultural University, Wuhan 430070, China; National Key Laboratory of Tropical Crop Breeding, Shenzhen Branch, Guangdong Laboratory of Lingnan Modern Agriculture, Key Laboratory of Synthetic Biology, Ministry of Agriculture and Rural Affairs, Agricultural Genomics Institute at Shenzhen, Chinese Academy of Agricultural Sciences, Shenzhen 518120, China; Xinjiang Key Laboratory of Biological Resources and Genetic Engineering, College of Life Science and Technology, Xinjiang University, Xinjiang, Urumqi 830046, China; National Key Laboratory of Tropical Crop Breeding, Shenzhen Branch, Guangdong Laboratory of Lingnan Modern Agriculture, Key Laboratory of Synthetic Biology, Ministry of Agriculture and Rural Affairs, Agricultural Genomics Institute at Shenzhen, Chinese Academy of Agricultural Sciences, Shenzhen 518120, China; Key Laboratory of Plant Resource Conservation and Germplasm Innovation in Mountainous Region (Ministry of Education), Institute of Agro-bioengineering/College of Life Sciences, Guizhou University, Guiyang 550025, China; National Key Laboratory for Germplasm Innovation & Utilization of Horticultural Crops, Zhengzhou Fruit Research Institute, Chinese Academy of Agricultural Science, Zhengzhou 450009, China; National Key Laboratory of Tropical Crop Breeding, Shenzhen Branch, Guangdong Laboratory of Lingnan Modern Agriculture, Key Laboratory of Synthetic Biology, Ministry of Agriculture and Rural Affairs, Agricultural Genomics Institute at Shenzhen, Chinese Academy of Agricultural Sciences, Shenzhen 518120, China; Department of Bioinformatics, School of Biology and Basic Medical Sciences, Suzhou Medical College of Soochow University, Suzhou 215123, China; National Key Laboratory of Tropical Crop Breeding, Shenzhen Branch, Guangdong Laboratory of Lingnan Modern Agriculture, Key Laboratory of Synthetic Biology, Ministry of Agriculture and Rural Affairs, Agricultural Genomics Institute at Shenzhen, Chinese Academy of Agricultural Sciences, Shenzhen 518120, China; College of Life Sciences, Wuhan University, Wuhan 430072, China; National Key Laboratory of Tropical Crop Breeding, Shenzhen Branch, Guangdong Laboratory of Lingnan Modern Agriculture, Key Laboratory of Synthetic Biology, Ministry of Agriculture and Rural Affairs, Agricultural Genomics Institute at Shenzhen, Chinese Academy of Agricultural Sciences, Shenzhen 518120, China; National Key Laboratory of Tropical Crop Breeding, Shenzhen Branch, Guangdong Laboratory of Lingnan Modern Agriculture, Key Laboratory of Synthetic Biology, Ministry of Agriculture and Rural Affairs, Agricultural Genomics Institute at Shenzhen, Chinese Academy of Agricultural Sciences, Shenzhen 518120, China; National Key Laboratory of Tropical Crop Breeding, Shenzhen Branch, Guangdong Laboratory of Lingnan Modern Agriculture, Key Laboratory of Synthetic Biology, Ministry of Agriculture and Rural Affairs, Agricultural Genomics Institute at Shenzhen, Chinese Academy of Agricultural Sciences, Shenzhen 518120, China; National Key Laboratory of Tropical Crop Breeding, Shenzhen Branch, Guangdong Laboratory of Lingnan Modern Agriculture, Key Laboratory of Synthetic Biology, Ministry of Agriculture and Rural Affairs, Agricultural Genomics Institute at Shenzhen, Chinese Academy of Agricultural Sciences, Shenzhen 518120, China; National Key Laboratory of Tropical Crop Breeding, Shenzhen Branch, Guangdong Laboratory of Lingnan Modern Agriculture, Key Laboratory of Synthetic Biology, Ministry of Agriculture and Rural Affairs, Agricultural Genomics Institute at Shenzhen, Chinese Academy of Agricultural Sciences, Shenzhen 518120, China; College of Horticulture and Forestry Sciences, Huazhong Agricultural University, Wuhan 430070, China; National Key Laboratory of Tropical Crop Breeding, Shenzhen Branch, Guangdong Laboratory of Lingnan Modern Agriculture, Key Laboratory of Synthetic Biology, Ministry of Agriculture and Rural Affairs, Agricultural Genomics Institute at Shenzhen, Chinese Academy of Agricultural Sciences, Shenzhen 518120, China; National Key Laboratory of Tropical Crop Breeding, Shenzhen Branch, Guangdong Laboratory of Lingnan Modern Agriculture, Key Laboratory of Synthetic Biology, Ministry of Agriculture and Rural Affairs, Agricultural Genomics Institute at Shenzhen, Chinese Academy of Agricultural Sciences, Shenzhen 518120, China; Key Laboratory of Plant Resource Conservation and Germplasm Innovation in Mountainous Region (Ministry of Education), Institute of Agro-bioengineering/College of Life Sciences, Guizhou University, Guiyang 550025, China; Institute of Pomology Science, Guizhou Academy of Agricultural Sciences, Guiyang 550006, China; National Key Laboratory of Tropical Crop Breeding, Shenzhen Branch, Guangdong Laboratory of Lingnan Modern Agriculture, Key Laboratory of Synthetic Biology, Ministry of Agriculture and Rural Affairs, Agricultural Genomics Institute at Shenzhen, Chinese Academy of Agricultural Sciences, Shenzhen 518120, China; National Key Laboratory of Tropical Crop Breeding, Shenzhen Branch, Guangdong Laboratory of Lingnan Modern Agriculture, Key Laboratory of Synthetic Biology, Ministry of Agriculture and Rural Affairs, Agricultural Genomics Institute at Shenzhen, Chinese Academy of Agricultural Sciences, Shenzhen 518120, China; National Key Laboratory of Tropical Crop Breeding, Tropical Crops Genetic Resources Institute, Chinese Academy of Tropical Agricultural Sciences, Haikou 570100, China; National Key Laboratory for Germplasm Innovation & Utilization of Horticultural Crops, Zhengzhou Fruit Research Institute, Chinese Academy of Agricultural Science, Zhengzhou 450009, China; Key Laboratory of Plant Resource Conservation and Germplasm Innovation in Mountainous Region (Ministry of Education), Institute of Agro-bioengineering/College of Life Sciences, Guizhou University, Guiyang 550025, China

## Abstract

The economically significant genus *Prunus* includes fruit and nut crops that have been domesticated for shared and specific agronomic traits; however, the genomic signals of convergent and divergent selection have not been elucidated. In this study, we aimed to detect genomic signatures of convergent and divergent selection by conducting comparative population genomic analyses of the apricot–peach–plum–mei (APPM) complex, utilizing a haplotype-resolved telomere-to-telomere (T2T) genome assembly and population resequencing data. The haplotype-resolved T2T reference genome for the plum cultivar was assembled through HiFi and Hi-C reads, resulting in two haplotypes 251.25 and 251.29 Mb in size, respectively. Comparative genomics reveals a chromosomal translocation of ~1.17 Mb in the apricot genomes compared with peach, plum, and mei. Notably, the translocation involves the *D* locus, significantly impacting titratable acidity (TA), pH, and sugar content. Population genetic analysis detected substantial gene flow between plum and apricot, with introgression regions enriched in post-embryonic development and pollen germination processes. Comparative population genetic analyses revealed convergent selection for stress tolerance, flower development, and fruit ripening, along with divergent selection shaping specific crop, such as somatic embryogenesis in plum, pollen germination in mei, and hormone regulation in peach. Notably, selective sweeps on chromosome 7 coincide with a chromosomal collinearity from the comparative genomics, impacting key fruit-softening genes such as *PG*, regulated by *ERF* and *RMA1H1*. Overall, this study provides insights into the genetic diversity, evolutionary history, and domestication of the APPM complex, offering valuable implications for genetic studies and breeding programs of *Prunus* crops.

## Introduction

Species were predominantly defined by reproductive isolation, as physiological and mating incompatibilities [1]. Nonetheless, many species are known to hybridize occasionally in captivity and the wild, accompanied by introgression (the acquisition of genetic variation from another species [2]), and recombinational speciation (today known as homoploid hybrid speciation). Phenotypic changes are primarily attributed to natural mutations, with occasional interspecific introgression [3, 4]. Though identifying genomic introgression is challenging, a wealth of genomic data confirms the occurrence of frequent gene flow between species, such as wheat [5], some of which are adaptive [6–10]. For instance, the domesticated grapevine (*Vitis vinifera*) has undergone additional genomic introgression from its wild relatives, resulting in enhanced fruit aroma and increased disease resistance [11]. Genomic regions with high recombination rates are more susceptible to introgression than regions with lower recombination rates [12], due to the effects of selection on sites linked to introgressed alleles (i.e. linked selection) [13, 14].

Such selection signals were mostly specific to given populations/species (divergent selection), but occasionally they could also be shared among populations/species (convergent selection). Convergent selection takes place when diverse species consistently encounter comparable selective pressures in their environments [15–17], whereas divergent selection results in trait divergence within a single lineage due to diverse selective pressures [18]. If a particular genomic region has positive (i.e. selective sweep [19]) or negative (i.e. purifying selection [20]) effects, both the region itself and neighboring sequences will show reduced genetic diversity. Selective sweeps identified in domesticated populations, including maize [21], cattle [22], grapes [23], and citrus [24], have enhanced our understanding of genetic architecture and artificial selection during the domestication history of agronomic traits [25, 26].

*Prunus* species are economically important plants in the subfamily Amygdaloideae of the Rosaceae [27], with genome sizes of ~230–280 Mb [28–30]. The genus includes a range of economically important flower, fruit, and nut crops [31, 32], in which apricot, peach, plum, and mei are closely related species with some levels of hybrid and grafting compatibilities among them. Historical gene flow was also reported, forming the apricot–peach–plum–mei (APPM) complex (2*n* = 2*x* = 16). The APPM complex includes four fruit tree species that share a more recent common ancestry within its constituent species compared with cherries [28–30]. Nevertheless, despite their historical significance and traditional classification, our knowledge of the genetic background of these complex remains very limited. *Prunus mume*, known as Chinese plum, mei, mume, Japanese plum, and Japanese apricot, is closely related to the apricot [33–37]. *Prunus brigantina*, resembling apricot in texture but with smooth skin like plums, is a wild species [38, 39]. *Prunus cerasifera* is crucial in polyploid breeding and is one of the parent species in the origin of *Prunus domestica* [40]. Furthermore, recent breeding programs have often utilized interspecific crossing of the subgenus *Prunus*, such as ‘Plumcot’ (*P. salicina* × *P. armeniaca*) [41, 42] or ‘Peacotum’ (*P. persica* × *P. armeniaca* × *P. salicina*) [43]. During the differentiation of these three species within the genus *Prunus* (*P. mume*, *P. armeniaca*, and *P. salicina*), several hybridization events may have occurred, resulting in significant introgressions associated with unidentified phenotypic changes during the formation of the common ancestor of *P. mume* [35]. The population genetics of the APPM complex can provide a deeper understanding of their convergent and divergent selection and domestication history [32, 44], which is vital for the process of *Prunus* breeding. However, the genomic signatures of convergent and divergent selection of favorable traits in these crops remain poorly understood.

To further explain the evolutionary history of *Prunus*, potential introgressions, and encompassing convergent and divergent selection, we explored comparative genomics and population genomics across different *Prunus* species, including apricot, peach, plum, and mei. Furthermore, we explored the domestication genomics of the APPM complex, thereby contributing novel perspectives to the realm of *Prunus* breeding strategies.

## Results

### Assembly of haplotype-resolved telomere-to-telomere reference genome of plum

**Figure 1:**
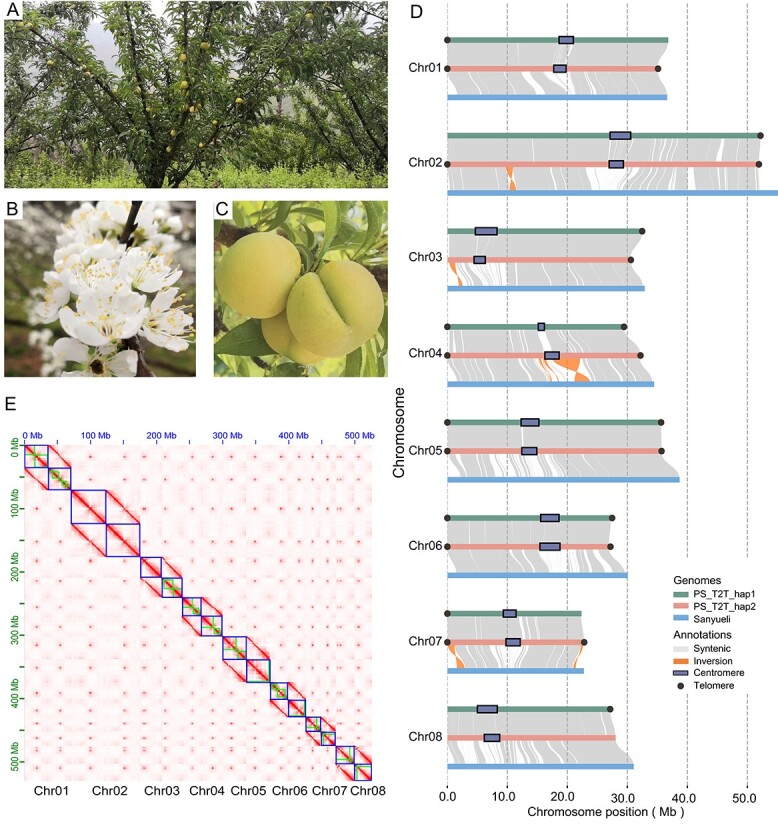
Overview of the PS_T2T reference genome. **A**–**C** The pictures used in this study show the tree, fruits, and flowers of the ‘Fengtangli’ plum. **D** Overview of genome assembly. Collinearity between ‘Sanyueli’ and two haplotypes of PS_T2T. Gray lines represent collinearity blocks with length 15 000 bp, while orange lines represent potential inversions. Centromeres and telomeres are indicated by black boxes and black dots, respectively. **E** Hi-C interaction matrix based on the PS_T2T diploid assembly.

We generated a total of 26.84 Gb (~100× coverage) of raw PacBio high-fidelity (HiFi) reads and 30 Gb (∼120× coverage) of chromosome conformation capture sequencing (Hi-C) data for assembling the plum (*P. salicina* cv. ‘Fengtangli’) genome (Fig. 1A–C, Supplementary Data Table S1). Before starting the assembly, we estimated genome heterozygosity to be 0.97% using a *k*-mer-based approach (Supplementary Data Fig. S1). For the initial assembly, the contig-level N50 values for haplotype 1 and haplotype 2 were 20.11 and 19.66 Mb, respectively, ~14 times that of the *P. salicina* cv. ‘Sanyueli’ contig-level assembly, with the largest contig reaching a length of 52.18 Mb. After anchoring and ordering, the scaffold N50 sizes reached 28.08 and 28.37 Mb. Benchmarking Universal Single-Copy Orthologs (BUSCO) assessment revealed that the conservative plant core genes are nearly fully captured at 99.2% for haplotype 1 and 98.9% for haplotype 2, marking an improvement from the previous rate of 98.64% (Table 1, Supplementary Data Fig. S1). Thirteen and 15 gaps were identified after initial assembly into the scaffold among two haplotypes (Supplementary Data Table S2). By mapping the HiFi data to each of the two haplotypes, we manually filled in all the gaps. Finally, we assembled two gap-free haplotypes of the *P. salicina* cv. ‘Fengtangli’, named PS_T2T_hap1 (251.25 Mb) and PS_T2T_hap2 (251.29 Mb) (Fig. 1D, Table 1). Visualization of Hi-C data using Juicebox [45] demonstrated a high level of consistency across all chromosomes for both haplotypes, proving their accuracy of ordering and orientation (Fig. 1E). By remapping the HiFi and Hi-C reads to their respective PS_T2T assemblies, we achieved a mapping ratio of >98.5%, emphasizing the accuracy and completeness of our genome assembly (Supplementary Data Table S1). In comparison, the quality of PS_T2T is similar to those of published complete genomes, including maize [46], rice [47], grapes [48, 49], pear [50], and kiwifruit [51]. These high-quality genomes provide the opportunity to study the evolutionary genomics among *Prunus* species.

**Table 1:** Statistics for *Prunus salicina* cv. ‘Fengtangli’ genome assembly and annotation.

**Genomic feature**	**PS_T2T_hap1**	**PS_T2T_hap2**	**Sanyueli**
Estimated genome size (Mb)	217.09	217.09	309.35
Assembled genome size (Mb)	251.25	251.29	282.38
Number of contigs ≥1 kb	269	165	502
N50 contig length (bp)	20 111 805	19 658 610	1 370 198
BUSCOs of contig	99.1	98.7	
Largest contig (bp)	52 178 471	51 917 112	8 384 049
Complete BUSCOs (%)	99.2	98.9	98.64
Complete and single-copy BUSCOs (%)	97.5	97	93.68
Complete and duplicated BUSCOs (%)	1.7	1.9	4.96
Fragmented BUSCOs (%)	0.6	0.7	0.37
Missing BUSCOs (%)	0.2	0.4	0.99
Number of gaps	0	0	536
Number of scaffolds	8	8	18
N50 scaffold length (Mb)	32.46	32.18	34.46
Mapping rate by reads from short-insert libraries (%)	99.8	99.8	96.6
Telomeres	11/16	13/16	0/16
Centromere	8/8	8/8	0/8
Number of genes	28 775	28 139	29 712
TEs (%)	45.41%	46.19%	

Centromeres are typically composed of repetitive DNA sequences, with variations among different organisms [52]. Tandem Repeats Finder (TRF) [53] found 470 different repeat units in the PS_T2T assemblies. Finally, 166-bp repeats were the most abundant unit in the whole genome. Transposable element (TE) analyses also supported the centromeric feature of the 166 bp repetitive unit (Supplementary Data Fig. S2). Therefore, the centromeres are primarily identified based on the 166-bp repeats and are located on all 16 chromosomes of the two haplotypes, with lengths ranging from 1.02 to 3.61 Mb in PS_T2T_hap1 and from 1.88 to 3.36 Mb in PS_T2T_hap2 (Supplementary Data Table S3). Telomere identification was performed by searching for telomere sequences. They consist of repetitive DNA sequences, such as CCCATTT at the 5′ end and TTTAGGG at the 3′ end in plants. Twenty-four of the expected 32 telomeres (8 chromosomes of two haplotypes) were identified, and 11 and 13 telomeres were found in PS_T2T_hap1 and PS_T2T_hap2 (Fig. 1D, Supplementary Data Fig. S3, Supplementary Data Table S4). Our PS_T2T assemblies filled in the absence of centromere and telomere assembly of the reference genome ‘Sanyueli’. Overall, the above results confirm a high-quality genome assembly.

Extensive *de-novo* TE Annotator (EDTA) [54] was used to generate a high-quality repetitive sequence library, and identified 119.63 and 121.71 Mb of repetitive sequences in the two haplotypes, accounting for 45.41 and 46.19% of PS_T2T_hap1 and PS_T2T_hap2, respectively. For haplotype 1, LTR retrotransposons account for 29.57%, which contained 18.07% Gypsy and 4.50% Copia LTR elements (Supplementary Data Table S5). For gene annotation, 28 775 and 28 139 protein-coding genes were predicted for the two haplotypes. BUSCO assessment using the longest transcribed proteins revealed that the two haplotypes captured 96.6 and 94.0% of a BUSCO reference gene set, respectively. Moreover, 40 270 and 3 205 transcripts were predicted with an average of 1.39 splice variants per gene. Additionally, 48 645 shared genes were obtained, including 24 542 from PS_T2T_hap1 and 24 103 from PS_T2T_hap2, belonging to 20 323 orthologous gene families, representing a core set of gene clusters in the PS_T2T genome (Supplementary Data Table S6). Furthermore, 876 genes were unique to PS_T2T_hap1, and 760 genes were unique to PS_T2T_hap2, likely reflecting genetic distinctions between the two parental strains (Supplementary Data Table S6). In conclusion, the depth of our sequencing and retention of more sequences resulted in a more comprehensive and accurate assembly of the diploid genome for the first time, representing a substantial advancement in plum genomics.

### Comparative genomics of the apricot–peach–plum–mei species

To further verify the quality of our assemblies, comparative genomics analyses were performed with two haplotypes using *P. salicina* cv. ‘Sanyueli’ as the reference genome (Supplementary Data Fig. S4). The results showed good collinearity in most regions of the genome. Non-collinearity might be caused by poor sequence quality, low coverage, or misassembly in the previous genome, but it could also be genuinely present, especially across the whole-genome scale. With a more complete diploid assembly of *P. salicina* cv. ‘Fengtangli’, we performed variant analysis between the two haplotypes. The comparison between the two haplotypes of PS_T2T indicates that they share a set of similar genomic features, including closed genome size, parallel repetitive content, and a comparable number of genes. However, a significant amount of variation is still observed between the two haplotypes, including 1 667 700 single-nucleotide polymorphisms (SNPs), around 389 962 insertions and deletions (InDels, <50 bp), and 30 698 structural variants (SVs, ≥50 bp [26, 55]). This establishes a substantial genetic diversity repository for plum (Supplementary Data Table S7, Supplementary Data Fig. S5). Some SVs, such as inversions, deletions, insertions, and translocations, were rarely observed in the newly assembled centromeric regions. This could be attributed to the presence of more conserved repeats and greater sequence stability within the highly repetitive mitotic satellites, as well as the lower diversity in mitotic patterns among haplotypes.

**Figure 2:**
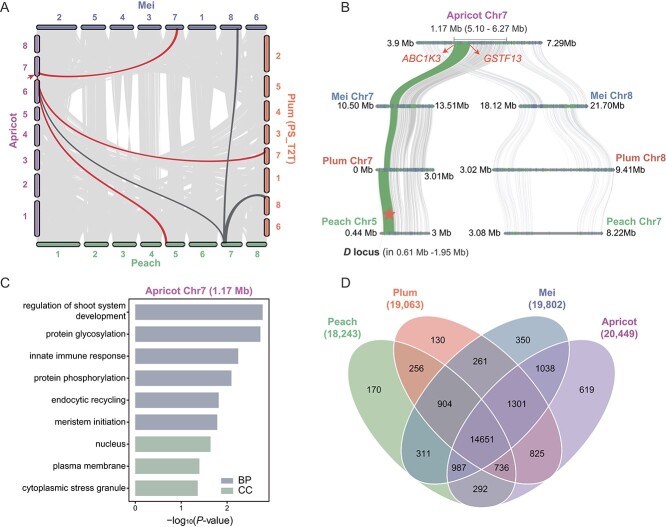
Comparative genomic analysis of APPM genomes. **A** Synteny analysis at the gene level among four *Prunus* species. Light gray represents syntenic blocks. Dark gray and red lines denote collinearity, with red arrows indicating a translocation. **B** Collinearity comparison of four genomes demonstrates apricot translocation spanning 1.17 Mb (Chr7: 5.10–6.27 Mb) shared with three other species. Green syntenic blocks indicate *D* loci associated with agronomic traits. **C** Top enriched biological processes for genes in the 1.17-Mb translocation region. **D** Venn diagram comparing shared and private gene families of four *Prunus* species.

To study the evolutionary conservation within the *Prunus* genus genome, we selected high-quality genomes (long-read based) from four species: apricot (*P. armeniaca*) [56], peach (*P. persica*) [57], plum (*P. salicina*, this study), and mei (*P. mume*) [58] for genome collinear comparison (Supplementary Data Table S8). For the plum, the two haplotypes we assembled exhibit a high degree of collinearity, and therefore we chose PS_T2T_hap1 as a representative of the plum species. A strong collinear relationship exists among the four *Prunus* species, indicating that our pseudo-chromosomes derived from anchored and oriented contigs were of high quality (Fig. 2A). For instance, chromosome 7 of apricot demonstrated significant collinearity with chromosome 7 in peach, chromosome 8 in plum, and chromosome 8 in mei.

A large structural rearrangement in the form of translocations was characterized among chromosome 7 in apricot compared with the others (Fig. 2A, Supplementary Data Table S9). However, an SV was detected in the apricot in the form of a translocation spanning the 5.10- to 6.27-Mb (1.17 Mb) region of the long arm of chromosome 7. This particular segment corresponded with chromosome 5 in peach, chromosome 7 in plum, and chromosome 7 in mei (Fig. 2B, Supplementary Data Table S10). Gene Ontology (GO) enrichment analysis indicates that the 1223 genes located in the translocation exhibit significant enrichment in various biological processes, including regulation of shoot system development, protein glycosylation, and innate immune response (Fig. 2C, Supplementary Data Table S11). At the breakpoint of the translocation, both genes on either side may be affected. These genes have been identified as *ABC1K3*, which is essential for the photo-oxidative stress response [59], and *GSTF13*, which plays a key role in the detoxification of certain herbicides [60] (Fig. 2B). We also highlighted the gene rearrangement events in the translocation region and identified a very important *D* locus associated with fruit flavor at the translocation of chromosome 5 in peach (Fig. 2B).

Additionally, a gene family cluster analysis was performed on the genomes of apricot, peach, plum, and mei. The results revealed that these four *Prunus* species collectively shared 14 651 gene families, and plum shared 2466 gene families with mei, 2862 with apricot, and 1896 with peach, with core genes being excluded from this analysis (Fig. 2D). Of these, 130 gene families, comprising 788 genes, were exclusively identified in the plum genome. The count is lower than that of the private genes noted in apricot (3450 genes across 619 gene families) and mei (1471 genes across 350 gene families) but exceeds that of peach, which had 299 genes within 107 distinct gene families (Fig. 2D, Supplementary Data Table S12). GO enrichment analysis showed that plum-specific genes were significantly enriched in biological processes such as signal transduction, receptor-mediated endocytosis, and defense response. Apricot-specific genes are mainly related to the regulatory sources of circadian rhythm and leaf senescence (Supplementary Data Table S13).

### Genetic diversity, divergence, and population structure in the apricot–peach–plum–mei complex

**Figure 3:**
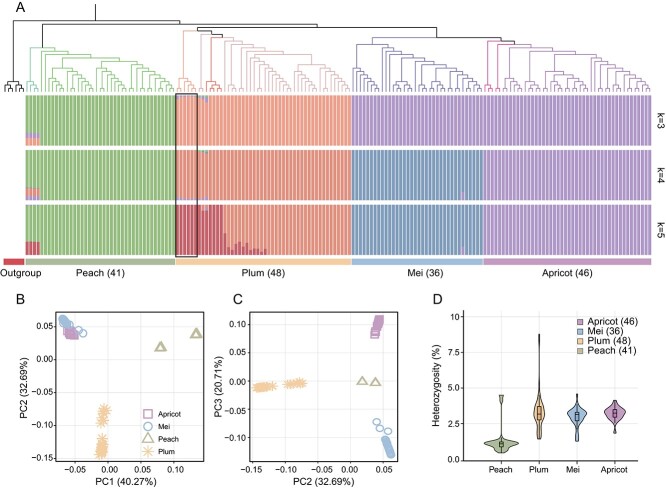
Population structure and heterozygosity of the APPM complex. **A** Phylogenetic tree of all accessions based on whole-genome SNPs. Different clades are represented by colored branches: 41 peach, 46 apricot, 36 mei, and 48 plum accessions. The estimated admixture proportions ranged from *K* = 3 to *K* = 5. **B**, **C** PCA of 171 samples in *Prunus*. **D** Heterozygosity in each species of the APPM complex.

To further study population genetics of introgression, and divergent and convergent selection in the APPM complex, whole-genome sequencing data were collected from 171 accessions, including 41 peach (4 *P. dulcis* and 37 *P. persica*), 46 apricot (5 *P. mandshurica* and 41 *P. armeniaca*), 36 mei (36 *P. mume*), 48 plum (1 *P. cerasifera* × *P. munsoniana*, 4 *P. cerasifera*, 6 *P. brigantina*, and 37 *P. salicina*), and 6 outgroups (3 apples and 3 pears) from worldwide sources (Supplementary Data Table S14). After mapping resequencing data to a reference genome, we filtered low-quality variants and retained a total of 8 011 197 high-quality SNPs. Based on the phylogenetic tree, we categorized the 171 accessions into four distinct clades: peach, plum, mei, and apricot accessions (Fig. 3A, Supplementary Data Fig. S6). We then investigated the population genetic structure from 2 to 15 clusters (*K*) based on 3 472 793 SNPs in 171 *Prunus* accessions to estimate the ancestral proportions of each germplasm (Fig. 3A, Supplementary Data Fig. S7). When *K* = 4, four clades were separated, but a small mixture of apricots was observed in the plum population. Principal component analysis (PCA) further confirmed these population affiliations, with samples from each clade clustered together (Fig. 3B and C, Supplementary Data Fig. S8). To explore the differences between these four clades, we assessed levels of population heterozygosity. Peach exhibited a lower population heterozygosity (1.33%) compared with the other populations: mei (2.95%), apricot (3.21%), and plum (3.31%) (Fig. 3D, Supplementary Data Table S14).

**Figure 4:**
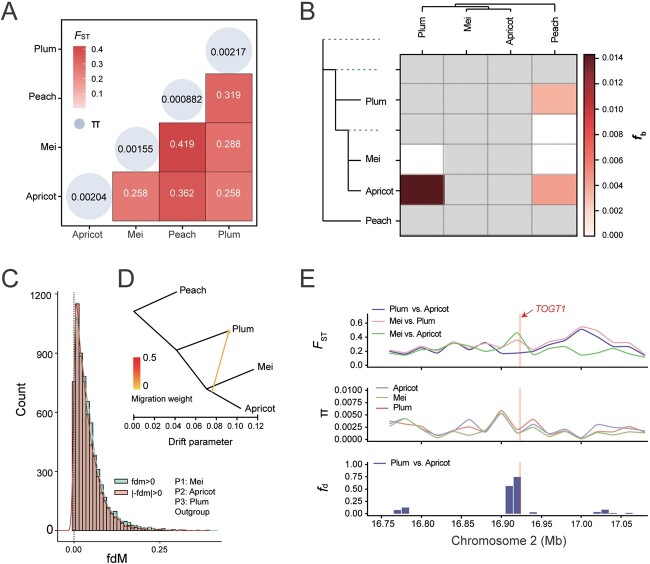
Genetic differentiation and extensive introgression among the four species in the APPM complex. **A** The heat map indicates the pairwise differentiation (*F*_ST_) statistics between the populations. Values within blue circles denote nucleotide diversity (π) for each population. **B** Heat map representation of *f_b_* statistics across different populations. Gray boxes correspond to pairs that could not be tested due to topological constraints occurring in the system. **C** Distribution of window counts based on the *f*_dM_ values for a 20-kb window. Green and pink colors represent the numbers of windows >0 and <0, respectively. **D** Inferred graphical representation highlighting migration events, based on genome-wide allele frequencies among the four clades. The yellow arrow indicates the direction of introgression. **E** Example of regions that are inferred to have introgressed from the apricot into plum. *F*_ST_, π, and *f_d_* values were evaluated in a 20-kb window. The prominently displayed red vertical line demarcates the region (Chr2: 16.92–16.94 Mb) believed to be under selection.

The fixation index (*F*_ST_) is a measure used in population genetics to quantify the level of genetic differentiation among populations. We calculated the values of *F*_ST_ and found that the highest *F*_ST_ was 0.419 between mei and peach, 0.362 between apricot and peach, and the lowest *F*_ST_, both 0.258 between apricot and mei and between apricot and plum. The results indicate a high genetic differentiation in mei and peach. To examine the levels of inbreeding in the four clades, we tested the genetic diversity (π). Among the three clades, peach exhibited the lowest nucleotide diversity (π = 8.82 × 10^−4^), followed by mei (π = 1.55× 10^−3^), apricot (π = 2.04 × 10^−3^), and plum (π = 2.17 × 10^−3^) (Fig. 4A, Supplementary Data Fig. S9).

### Gene flow in the apricot–peach–plum–mei complex

The *F*_ST_ and admixture results suggest potential genetic information exchange between plum and apricot, but the specific pattern of gene flow is not clear (Figs 3A and 4A). To determine possible genome-wide introgression in present species clades, we estimated the F-branch (*f_b_*) statistics using Dsuite [61] to quantify potential signals of related gene flow and patterns of allele-sharing specific to branches. Peach had a weaker introgression signal with plum (*f_b_* = 0.007, *Z* = 3.93) and apricot (*f_b_* = 0.004, *Z* = 3.31). Strong signals of introgression (*f_b_* = 0.014, *Z* = 7.42) were identified in the plum and apricot (Fig. 4B), suggesting potential introgression events among these species. As with *f_b_*, the triple topology showed that the apricot–plum comparison (average *f_d_* of 7.02 × 10^−2^) had higher genome-wide values than the apricot–peach comparison (average *f_d_* of 5.78 × 10^−2^, Supplementary Data Fig. S10, Supplementary Data Table S15). Given the evidence for introgression, we also applied the *f*_dM_ and *f_d_* statistics based on 20-kb (overlapping 10-kb) windows. The *f*_dM_ statistic and the histogram confirm a high level of introgression between plum and apricot, as there are 6463 windows (20 kb) between plum and apricot, and 6057 windows between mei and plum (Fig. 4C, Supplementary Data Tables S15 and S16).

To clarify the direction of gene flow migration between the plum and apricot, we inferred migration events and patterns based on whole-genome allele frequency data. TreeMix [62] results suggest a migration event between plum and apricot, sharing crucial signals from specific genotypes (*m* = 1, 2), which is associated with a lower migration weight (Fig. 4D). In addition, we analyzed genes within the top 5% of high *f_d_* windows between apricot and plum, identifying a total of 524 genes (Supplementary Data Table S17). GO analysis indicated that these genes were notably enriched in biological processes, including cysteine biosynthetic process, regulation of post-embryonic development, cell wall organization, and pollen germination (Supplementary Data Fig. S11, Supplementary Data Table S18). Furthermore, we inscribed the genome-wide region of the highest *f_d_* potential introgression between apricot and plum (Chr2: 16.92–16.94 Mb). As expected, π, population branch statistics (PBS) (Supplementary Data Fig. S12), and *F*_ST_ in apricot and plum populations were much lower in this region (Fig. 4). It is worth noting that the top of the *f_d_* outlier window contained introgressed signals related to the *TOGT1* gene on chromosome 2 (Chr2: 16922546-16 923 985 bp). This gene is associated with phenylpropanoid glucosyltransferase, which leads to the reduction of scopoletin glucoside accumulation, enhancement of oxidative stress, and weakening of virus resistance [63].

### Convergent and divergent selection signals in the apricot–peach–plum–mei complex

**Figure 5:**
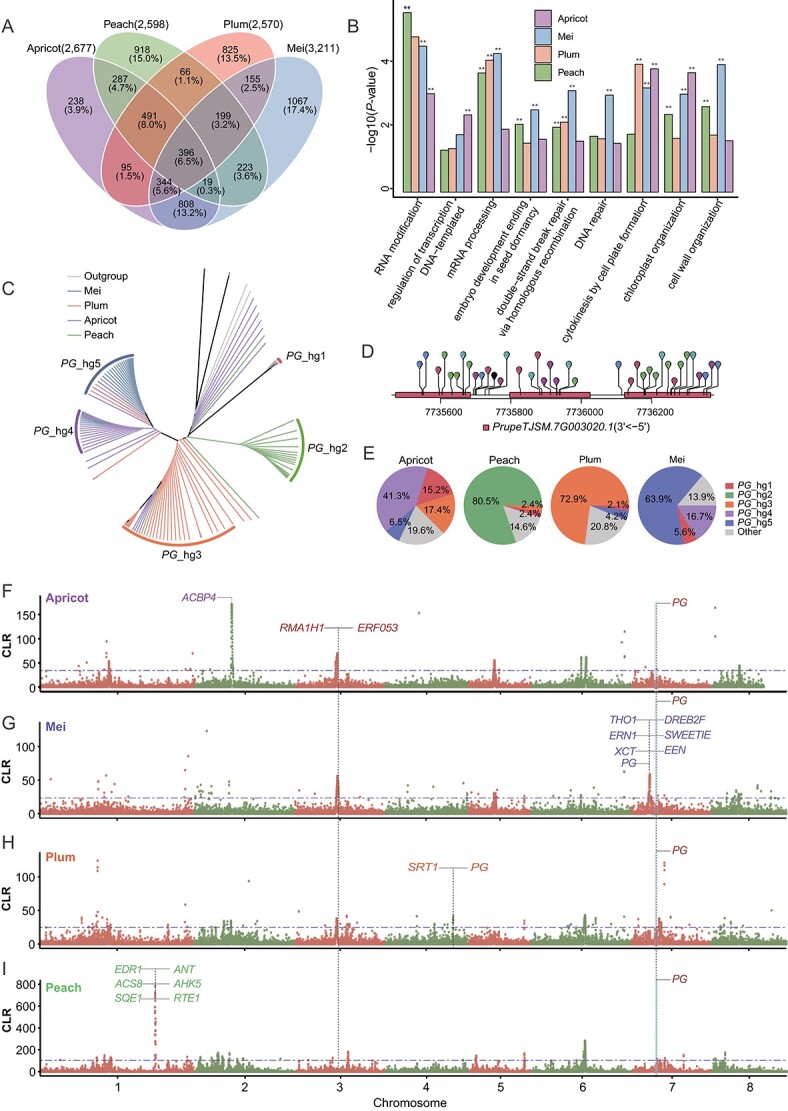
Convergent and divergent selection signals in the APPM complex. **A** Venn diagram of genes calculated to be in the top 1% CLR regions detected by SweeD in apricot, mei, plum, and peach. **B** Shared GO terms enriched in the top 1% CLR regions detected by SweeD. GO terms with a *P*-value <0.01 (Fisher’s exact test) are indicated with two asterisks. **C** Phylogenetic tree of functional haplotype sequences in highlighted genes shared across four clades: chromosome 7 (*PG*). **D** Visualization of variant positions above the *PG* gene model: the black line represents the genome and rectangles represent exons. **E** Population frequencies of *PG* haplotype clades in the APPM complex. **F**–**I** The dashed lines mark the regions in the top 1%. The red italicized text indicates functional genes common to all four clades, while purple, blue, orange, and green represent functional genes unique to apricot, mei, plum, and peach, respectively.

To examine the potential mechanism of adaptation to environmental changes during *Prunus*’s evolution, we performed a composite likelihood ratio (CLR) test and assessed the evidence for positive selection in different populations [56, 64]. We compared the genomes of 41 peach, 46 apricot, 36 mei, and 48 plum accessions to detect selective sweeps [23, 64] Our analysis pinpointed genes within the top 1% CLR regions identified by SweeD in apricot, mei, plum, and peach (Fig. 5A, Supplementary Data Table S19). Subsequently, we performed GO enrichment analysis for genes within these top 1% CLR regions and discovered nine functional categories that were shared among all four clades (*P* ≤ 0.01), including RNA modification, embryo development ending in seed dormancy, cytokinesis by cell plate formation, and cell wall organization (Fig. 5B, Supplementary Data Table S20). We identified common genes shared across four clades and genes unique to individual clades. Shared genes located on chromosomes 1, 3, 6, and 7 include those involved in response to biological and abiotic stress (e.g. *EP3*, *BAGP1*, *TIFY9*), flower development and seed-related processes (e.g. *TCP4*, *SPL4*, *TIO*), and fruit ripening (e.g. *PG*) (Supplementary Data Fig. S13A–D, Supplementary Data Table S21). Apricot-specific genes on chromosome 2 are enriched in processes such as callose deposition [65], chloroplast RNA processing, and anther wall tapetum development (e.g. *PME5*, *BHLH10*, *BHLH91*). Mei-specific genes on chromosome 7 are associated with pollen germination, vesicle fusion, growth regulation, and carbohydrate metabolism (e.g. *SWEETIE*, *NDF5*, *BDG4*) [66–68]. Plum-specific genes on chromosome 4 are linked to plastid and chloroplast fission, and somatic embryogenesis. Peaches exhibit specific genes on chromosome 1, including auxin polar transport and hormone signal regulation genes (e.g. *3BETAHSD/D2*, *ACL5*, *APM1*, *AHK5*, *AIP2*, *GCR1*) (Supplementary Data Fig. S16A and F, Supplementary Data Tables S24 and S25).

Our analyses detected genomic regions associated with convergent selectivity sweeps. Specifically, we identified that the convergent region on chromosome 7 corresponds to a chromosomal collinearity in peach according to comparative genomics analysis (Fig. 5F, Supplementary Data Fig. S14, Supplementary Data Tables S21 and S22). Evolutionary tree analysis of SNPs within the convergent region on chromosome 7 was consistent with the analysis utilizing all 3 472 793 SNPs (Supplementary Data Fig. S13E, Fig. 3A). Further examination focused on individual genes within this region, particularly Polygalacturonase (*PG*), which exhibited a distinct evolutionary tree compared with others. Given *PG*’s known association with fruit ripening and softening, it might be regulated by two convergent genes [69, 70]: E3 ubiquitin-protein ligase (*RMA1H1*) and Ethylene-responsive transcription factor (*ERF053*) (Fig. 5F). The evolutionary tree results for these genes were largely consistent with the analysis using all 3 472 793 SNPs, thereby indicating the significance of *PG* for further exploration (Supplementary Data Fig. S13F–H). The analysis of haplotypes for all coding regions of the PG gene generated a diverse array of haplotypes (Supplementary Data Fig. S15, Supplementary Data Table S23). A total of 60 haplotypes were identified, which were categorized into five haplotype clades (*PG*-hg1/2/3/4/5) based on a neighbor-joining tree (Fig. 5C, D). These clades exhibited non-uniform distribution across the APPM populations (Fig. 5E), with distinctive proportions observed in different fruit types.

Additionally, we identified unique genes under divergent selection in the top 1% CLR regions in apricot, mei, plum, and peach populations. Mei possesses two specific *PG* genes, while plum has one unique *PG* gene. In the AP2/ERF transcription factor family, mei harbors two distinct genes, and peach has one exclusive gene. Regarding the response to ethylene, apricot (*ACBP4*) has one unique gene, and peach (*SQE1*) possesses another. In the regulation of the ethylene biosynthetic process, mei (SWEETIE) possesses one exclusive gene, while peach (ACS8) also harbors one. For the ethylene-activated signaling pathway, mei (*EEN*, *THO1*, *XCT*) has three unique genes, plum (*SRT1*) has one, and peach (*AHK5*, *EDR1*) has two. Notably, peach has two genes dedicated to the negative regulation of the ethylene-activated signaling pathway (*AHK5*, *RTE1*). *AHK5* plays a dual role in both the ethylene-activated signaling pathway and its negative regulation (Fig. 5F–I, Supplementary Data Fig. S16, Supplementary Data Table S26).

In conclusion, our comprehensive analysis provides valuable insights into the genetic variations and associations within key genomic regions related to fruit ripening and ethylene response, paving the way for further investigation into the functional roles of these genes in fruit development and quality regulation.

## Discussion

The APPM complex shares a recent evolutionarily history and convergent and divergent signals of selection. Candidate genes could be applied in genetic studies and breeding programs for the APPM crops. In this research, by integrating HiFi and Hi-C reads, the haplotype-resolved T2T reference genome of plum was successfully assembled for the first time. The average N50 value of this genome is 19.85 Mb, which is 14 times higher than previously published genomes [30, 71]. Comparative genomics enables the identification of shared and unique genomic regions across *Prunus* species, revealing patterns of gene acquisition, loss, and duplication that underlie their adaptive strategies [72, 73]. Through genome collinearity analysis, a strong shared relationship was evident among the genomes of *Prunus* species [28–30]. Significantly, this study reports chromosomal translocation of ~1.17 Mb (Chr7: 5.10–6.27 Mb) for the first time, revealing structural changes in the genomes of apricot compared with peach, plum, and mei [56, 58]. The acidity and sugar of peach fruit are controlled by *D* locus, which is short for ‘doux’, meaning ‘sweet’ in French [74–77], and is mapped to a 509-kb interval on Chr5: 703–1212 kb [78]. Over the past several decades, it has remained a captivating subject of genetic investigation. Additionally, as we explore the analysis of the *D* locus in *Prunus* species, the translocation (Chr5: 611362–1 950 661 bp) in peach merits consideration (Fig. 2B, Supplementary Data Tables S9–S11).

Phylogenetic and population structure analyses suggest a close shared ancestry among plum, apricot, and mei, with the possibility of independent evolution driven by specific environmental or anthropogenic factors during the evolutionary process. Our findings indicate that *P. brigantina* is aligned with the plum category, as supported by chloroplast DNA sequences [39], but diverges from this alignment in nuclear DNA sequences, being closer to apricot [38], suggesting it might be a hybrid descendant of both plum and apricot species, with the admixture results reflecting the influence of different parental species (Fig. 3A–C, Supplementary Data Figs S6 and S7, Supplementary Data Table S14). Peaches exhibit notably lower heterozygosity values (Fig. 3D). Historically, peaches have been associated with reduced genetic variability due to their self-compatible (SC) mating system [79, 80]. However, in the *Prunus* genus, self-incompatibility (SI) is generally the rule, with most species being partially or fully self-incompatible, thereby facilitating gene flow [66, 81]. For introgression to take place, two species must have overlapping geographic ranges and must be sufficiently closely related so that at least some hybrid offspring are fertile. Despite most hybrids being unfit, some recombinant hybrids must have high fitness in the adaptive landscape. The observed high diversity in the *Prunus* genus could also be attributed to interspecific hybridization and additional introgression through backcrossing among closely related *Prunus* species [40, 82].

Our findings present compelling evidence of genetic exchange between plum and apricot, which contradicts previous studies [38]. According to speciation with gene flow models, a key prediction is that loci involved in speciation should exhibit high relative divergence (*F*_ST_) [83–85]. Notably, the lowest *F*_ST_ value (0.258) was observed between apricot and mei and between apricot and plum, indicating a high level of gene flow between apricot and either mei or plum. As seen in many other species, such as three-spined sticklebacks, regions of the genome with high rates of recombination facilitate the decoupling of deleterious introgressed alleles from neighboring loci, promoting gene flow and resulting in lower levels of divergence (*F*_ST_) in these regions [1, 86]. Mei displays high genetic diversity, originating from independent domestication events, and a relatively small proportion of the genome is affected by selection [56]. The *f_b_* statistic results reflected no evidence for introgression between the apricot and mei clades (*f_b_* = 0), but the *f_b_* value (0.014) between the apricot and plum clades was consistent with the possibility of introgression (low *F*_ST_). We have utilized ABBA-BABA statistics (e.g. *f_b_*, *f_d_*, and *f*_dM_) and TreeMix to estimate potential migration events, revealing ample evidence for introgression from apricot to plum (Fig. 4C and D, Supplementary Data Tables S15 and S16). Given the introgression between apricot and plum, an important question is whether it has had an adaptive basis. Adaptive introgression has played a significant role in crop domestication, as observed in crops like maize, barley, potato, and rice [87]. Our analyses offer compelling evidence that introgression has contributed to essential agronomic traits in plums, with the introgression enriched with 524 pivotal genes related to important biological processes, including the regulation of postembryonic development and pollen germination (Supplementary Data Tables S17 and S18). Studies conducted in other taxa have also demonstrated that introgression can promote species adaptation through various mechanisms [1, 88].

The APPM complex shares a recent evolutionary history, and it is essential to uncover the convergent and divergent signals of genomic selection in their genomes during domestication and adaptation for interspecific hybrid breeding. In our study, we identified a series of shared and specific genes among these four clades, revealing the complex genetic landscape during their evolution and adaptation. Each gene plays a distinct functional role in response to various environmental cues and developmental processes (Fig. 5, Supplementary Data Figs S13 and S16). Notably, convergent selectivity sweeps on chromosome 7 coincide with a chromosomal collinearity from the comparative genomics (Supplementary Data Tables S21 and S22), impacting crucial fruit softening genes like *PG*. The regulatory network involves specific genes like *MdPG1* and *FcPG12*, regulated by ERFs such as *MdCBF2* and *FcERF5*, contributing to fruit softening [69, 70]. CRISPR/Cas9 applications in tomatoes demonstrate the potential for delaying fruit softening [89]. The ubiquitin-mediated proteolysis pathway, represented by E3 ligases like *PRT6*, influences gene expression, impacting ERF regulation [90]. The transcriptional repressor *SlERF.F1*2, known for recruiting co-repressors and histone deacetylases, influences fruit ripening in tomatoes. These findings emphasize the complex molecular mechanisms governing fruit development and postharvest traits [69, 70, 89–91]. Additionally, the identification of a peach-specific divergent selection region on chromosome 1 [92], influenced by domestication and associated with increased fruit size, provides valuable insights into the transition from wild to cultivated varieties in *Prunus* crops. These candidate genes hold potential for future genetic studies and breeding programs, contributing to ongoing improvements in *Prunus* characteristics.

The potential for hybridization and introgression between two populations represents a multifaceted process shaped by genetic divergence, selection, recombination, and demographic factors. Adaptive introgression of a specific allele depends on its advantages, genomic location, impact on recipient population fitness, and recent demographic history [1].

The outcomes of hybridization are predominantly determined by long-term influences, including the fitness of early-generation hybrids and the emergence of novel allele combinations, which serve as catalysts for evolutionary change by introducing fitness variability. Under variable environmental conditions, this process enables populations to overcome challenges, explore new evolutionary paths, and persist.

## Materials and methods

For full materials and methods, see Supplementary Data.

### Plant materials and sequencing

*Prunus salicina* cv. ‘Fengtangli’ is a high-quality plum variety. Plant materials were collected from Guizhou Province. The Hi-C library was prepared with the NEBNext^®^ Ultra™ II kit and sequenced on an Illumina HiSeq X Ten (150PE). Two replicates per group ensured robust Hi-C results. For HiFi sequencing, PacBio Sequel II with CCS was used, and SMRTlink software processed raw data for high-quality sequences, adhering to specific quality parameters.

### Genome *de novo* assembly and quality assessment

The HiFi and Hi-C data were assembled using hifiasm [93, 94], producing two contig-level haplotype genomes. Genome heterozygosity was estimated using a *k*-mer-based approach by GenomeScope [95]. RagTag [96] anchored and removed short contigs, aligning with *P. salicina* cv. ‘Sanyueli’ serving as the reference genome. Juicer [97] and 3D-DNA [98] were employed for scaffold-level assembly by Hi-C data. For better genome quality, the obtained results were manually adjusted using Juicebox [45] before running 3D-DNA again to obtain the genome at the scaffold level. To verify the correctness of gap filling, Minimap2 [99] was used to compare the original HiFi data for sequence comparison. The resulting sequence positions of gaps were located using IGV [99], and the gaps were subsequently filled in the genome using Minimap2. Genome completeness relied on BUSCOs with the embryophyta_odb10 database [100], and continuity was assessed via contig N50 length.

### Identification of telomeres and centromeres

To identify telomeres, we used TIDK to recognize telomeric sequences with CCCATTT at the 5′ end and TTTAGGG at the 3′ end. To detect centromeric regions, we scanned candidate repeats from 30 to 500 bp along the genome using TRF v4.09 [53]. Then, the centromeric repeat units were identified by comparing the results of the TE distribution. The 166-bp repeats were the most abundant unit in the whole genome.

### Genome annotation and identified transposable elements

The raw RNA sequence data were trimmed with Trimmomatic [101] to eliminate adapter sequences. HISAT2 [102] and StringTie [103] were employed to remove bases with >10% ambiguous nucleotides (N) and quality values ≤5. Gene annotation followed the Genome-Wide Annotation Pipeline (https://github.com/unavailable-2374/Genome-Wide-Annotation-Pipeline). Functional gene annotation was conducted using InterProScan [104].

### Comparative genomics and gene families

For whole-genome alignment, Minimap2 was used to align the genomes, and the BAM file was indexed using SAMtools [105]. We used SyRI [106] for genome collinearity alignment and Plotsr for visualization. Additionally, we aligned the PS_T2T genome to the *P. salicina* cv. ‘Sanyueli’ reference using MUMmer, extracted detailed comparisons with the parameter show coords -T -q -H, and created dot plots for visualization with Gnuplot.

MCscan (Python version) was used for collinearity analysis of apricot [56], peach [57], plum, and mei [58] genomes and detection of structural changes. We used Orthofinder to analyze the gene families of apricots, peaches, plums, and plums [107]. David was used for GO enrichment analysis on shared and species-specific gene sets.

### Phylogenetic and population structure analyses

We analyzed illumina raw reads from 177 samples (SRA at NCBI) (Supplementary Data Table S14). Illumina short reads were aligned to the TJSM genome using BWA-MEME and sorted with SAMtools [105], and PCR duplicates were removed with GATK [108]. SNPs were called and filtered using GTX [109, 110] and VCFtools [111], leaving 3 749 618 SNPs for heterozygosity analysis, calculated as (N(NM) − O(HOM))/N(NM), where N(NM) represents observed variants and O(HOM) represents observed homozygous variants [112]. PLINK v1.9 [113] was used for a phylogenetic tree, population structure, and PCA on samples with MAF ≥0.05 and missing rate ≤20%. FastTree2 [114] built the phylogenetic tree, and ADMIXTURE [115] explored population structure (*K* from 2 to 15). Heterozygous sites and π for SNPs in each group were computed using VCFtools v0.1.15. Fixation indices (*F*_ST_) were calculated with 50-kb non-overlapping windows to identify domestication and differentiation regions.

### Introgression analysis with ABBA-BABA tests and TreeMix

We analyzed introgression events using *D*-statistic, *f_d_*, *f*_dM_, and *f_b_* statistics with a dataset of 8 011 997 SNPs . For a triplet of taxa P1, P2, and P3, and an outgroup, which follows the phylogeny of (((P1, P2), P3), Outgroup), a *D*-statistic significantly different from zero indicates P3 exchanged genes with P1 (*D* value 0) or P2 (*D* value >0). To assess introgression divergence, we filtered windows with <100 genotyped variants, computing *f_d_* and *f*_dM_ using ABBABABAwindows.py. *D*-statistics based on SNP frequency differences were also computed to identify gene exchange. TreeMix [62] inferred population relatedness and migration events, highlighting results using the plotting_func.R script. PBS was calculated using PBScan, each with 20-kb sliding windows, and windows with the top 5% of values were selected as highly divergent regions.

### Detection genome scanning for selective sweep signals

We used SweeD [64] with default parameters to calculate CLR scores, identifying potential regions under positive selection in peach, apricot, plum, and mei. The top 1% regions of CLR scores were considered as potential positive selection, and gene functions within these regions were annotated. Heterozygous sites, π, and *F*_ST_ were calculated with VCFtools v0.1.15 in 50-kb non-overlapping windows. PBS was calculated using PBScan, each with 20-kb sliding windows, and windows with the top 5% of values were selected as highly divergent regions.

## Acknowledgements

We are grateful to the Agricultural Genomics Institute at Shenzhen, Chinese Academy of Agricultural Sciences for providing the high-performance computing platforms during this study. This work was financially supported by the Guizhou Provincial Science and Technology Projects of China ([2022]Zhongdian018, YQK[2023]008), the National Guidance Foundation for Local Science and Technology Development of China (Grant No. 2023-009), and the Science Fund Program for Distinguished Young Scholars of the National Natural Science Foundation of China (Overseas) to Yongfeng Zhou. We also thank all members of the Zhou Lab for their valuable discussions and constructive comments.

## Author contributions

G.Q., K.C., and Y.Z. were responsible for conceiving and designing the research. X.Y., Y.S., and S.H. contributed to writing the manuscript. X.Y. and S.H. were responsible for collecting and updating the genome assemblies, while Y.S. performed the gene annotation. All authors contributed to the data collection and/or bioinformatics analyses. Y.M. and Q.H. provided the experimental materials. All authors have reviewed and approved the final manuscript.

## Data availability

The PS_T2T raw genome sequencing reads were available from the NCBI under project ID PRJNA1000098. All raw sequence data and assembly have been deposited in the National Genomics Data Center (NGDC) Genome Sequence Archive (GSA) (https://ngdc.cncb.ac.cn/gsa/), with BioProject number PRJCA018700. The assembly and annotation have been deposited in zenodo: https://zenodo.org/records/10570999.

## Conflict of interest

The authors declare that they have no competing interests.

## Supplementary data

Supplementary data are available at *Horticulture Research* online.

## Supplementary Material

Web_Material_uhae109
