# Supplementary material for: Comparative population genomics reveals convergent and divergent selection in the apricot–peach–plum–mei complex
Source: Hortic Res. 2024 Apr 16;11(6):uhae109. doi: 10.1093/hr/uhae109 (PMC11179850; doi:10.1093/hr/uhae109)
Supplement: Web_Material_uhae109 [file web_material_uhae109.zip › FigS_uhae109.pdf]

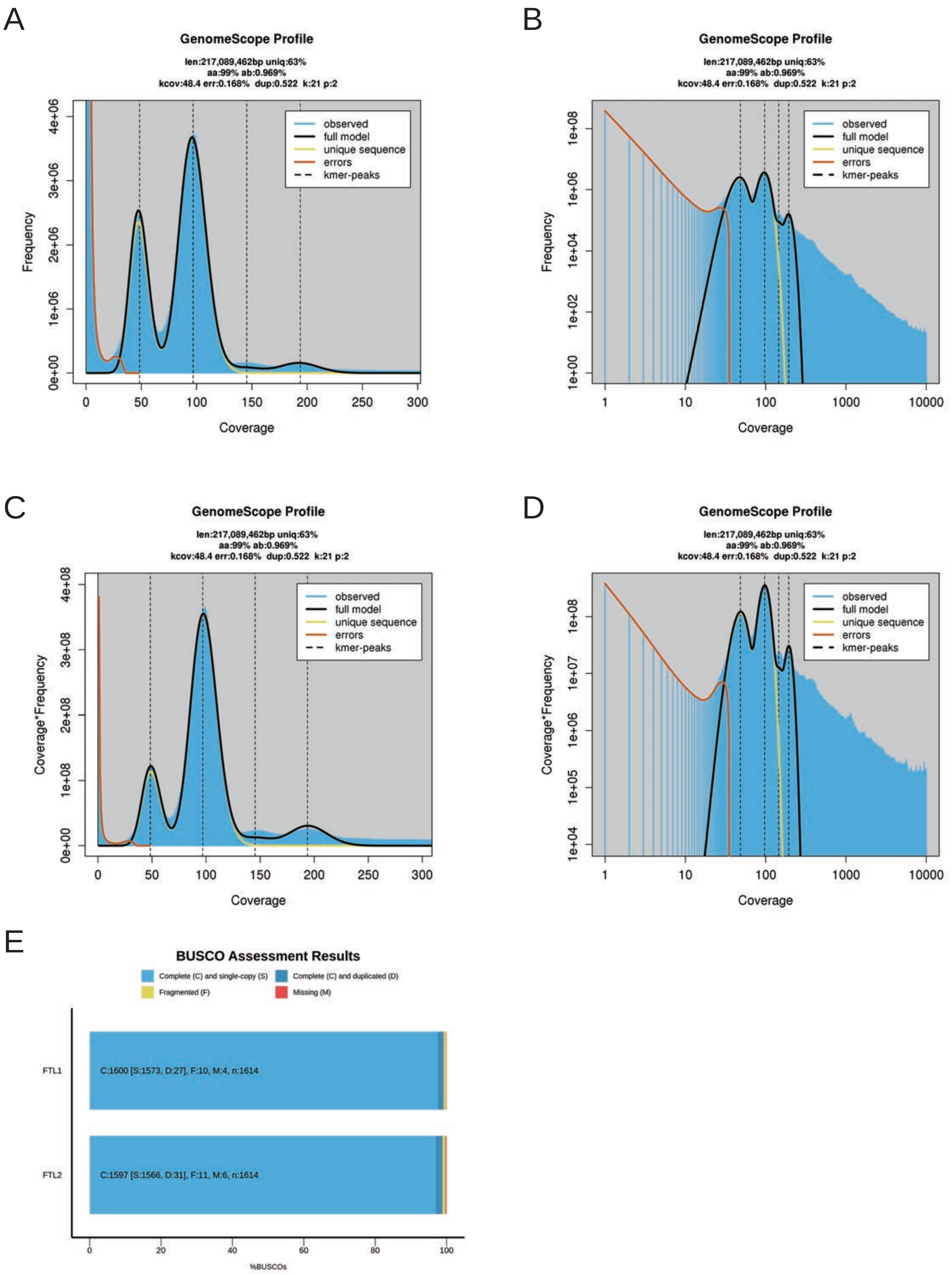

Figure S1 Assessment of genome heterozygosity by K-mer and BUSCOs. (A-D) Estimation of genome size based on 21 K-mer distribution. (E) BUSCO results for two genomes assembled.

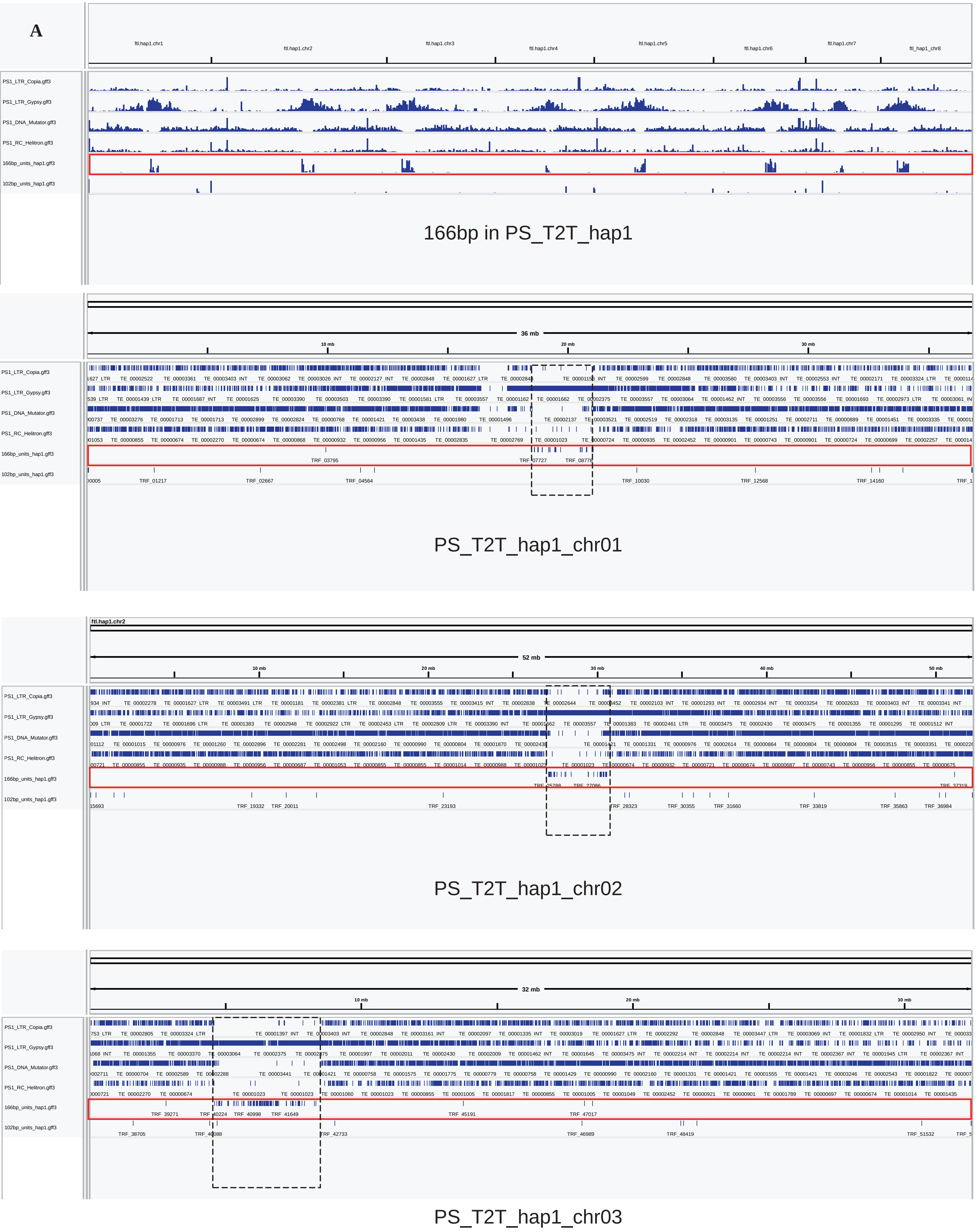
A
ftl.hap1.chr1
ftl.hap1.chr2
ftl.hap1.chr3
ftl.hap1.chr4
ftl.hap1.chr5
ftl.hap1.chr6
ftl.hap1.chr7
ftl_hap1_chr8
PS1_LTR_Copia.gff3
PS1_LTR_Gypsy.gff3
PS1_DNA_Mutator.gff3
PS1_RC_Helitron.gff3
166bp_units_hap1.gff3
102bp_units_hap1.gff3
166bp in PS_T2T_hap1
36 mb
10 mb
20 mb
30 mb
PS_T2T_hap1_chr01
ftl.hap1.chr2
52 mb
10 mb
20 mb
30 mb
40 mb
50 mb
PS_T2T_hap1_chr02
32 mb
10 mb
20 mb
30 mb
PS_T2T_hap1_chr03

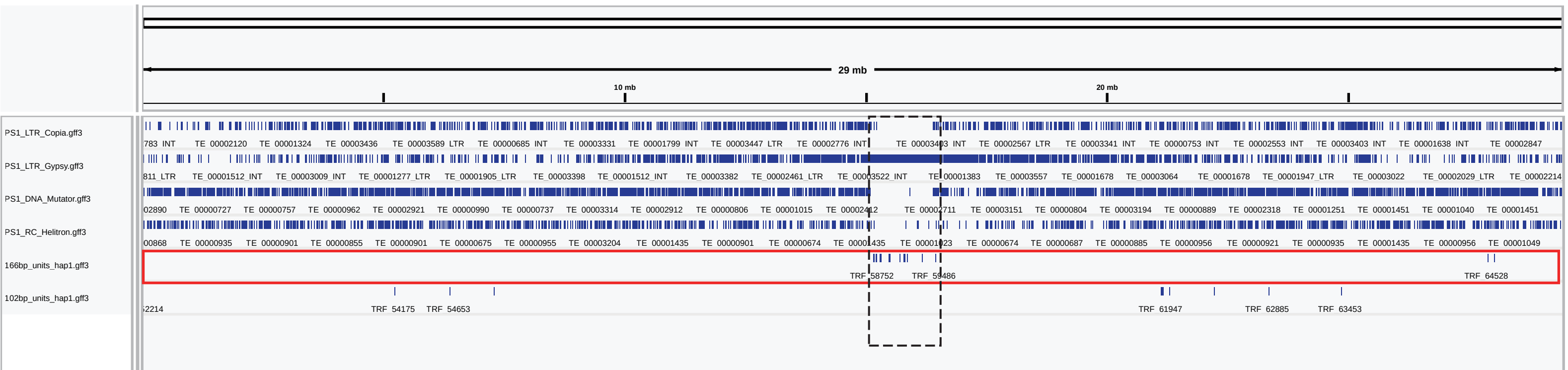

PS_T2T_hap1_chr04

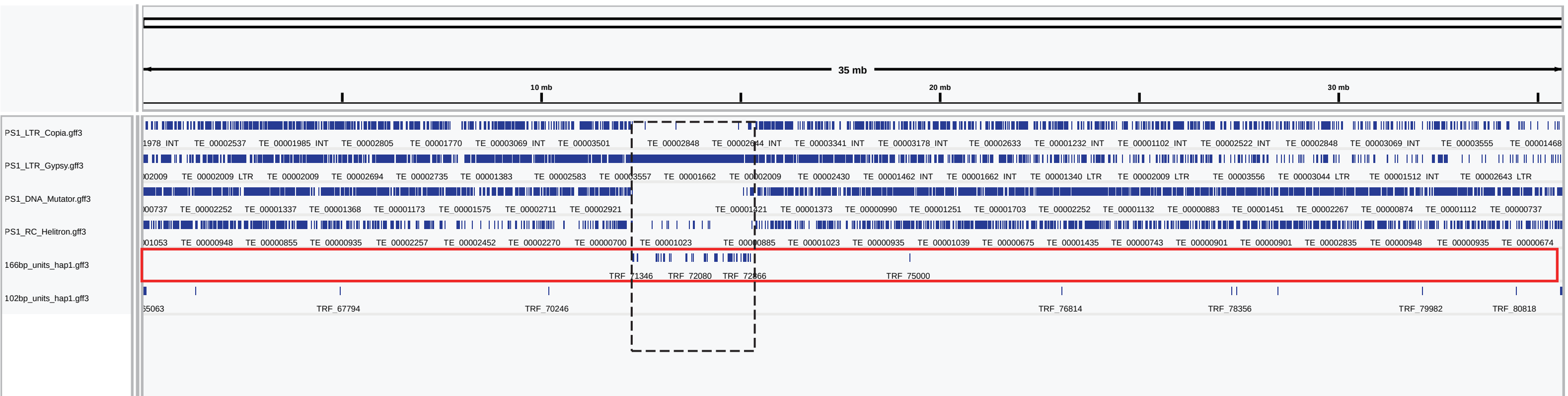

PS_T2T_hap1_chr05

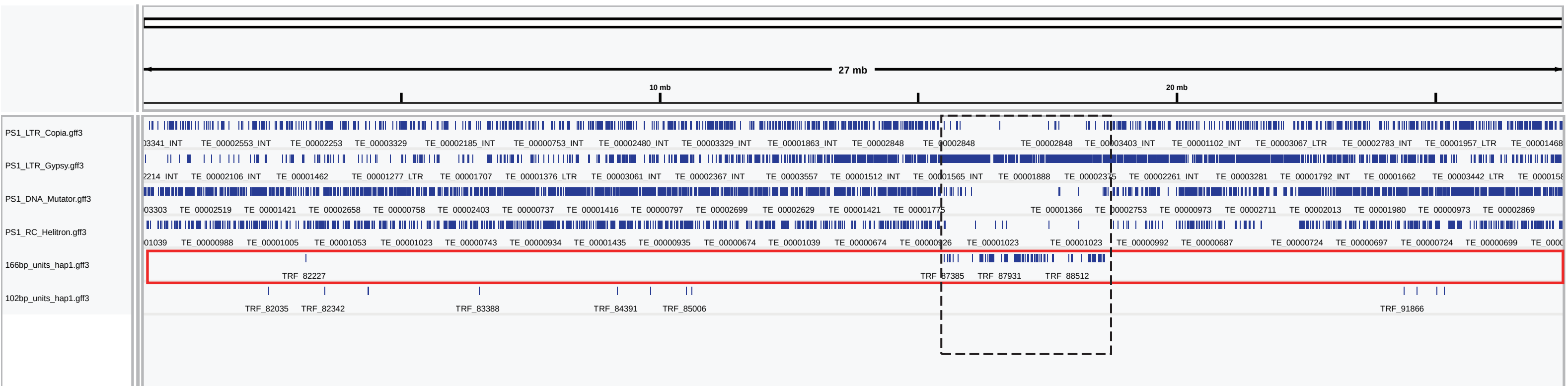

PS_T2T_hap1_chr06

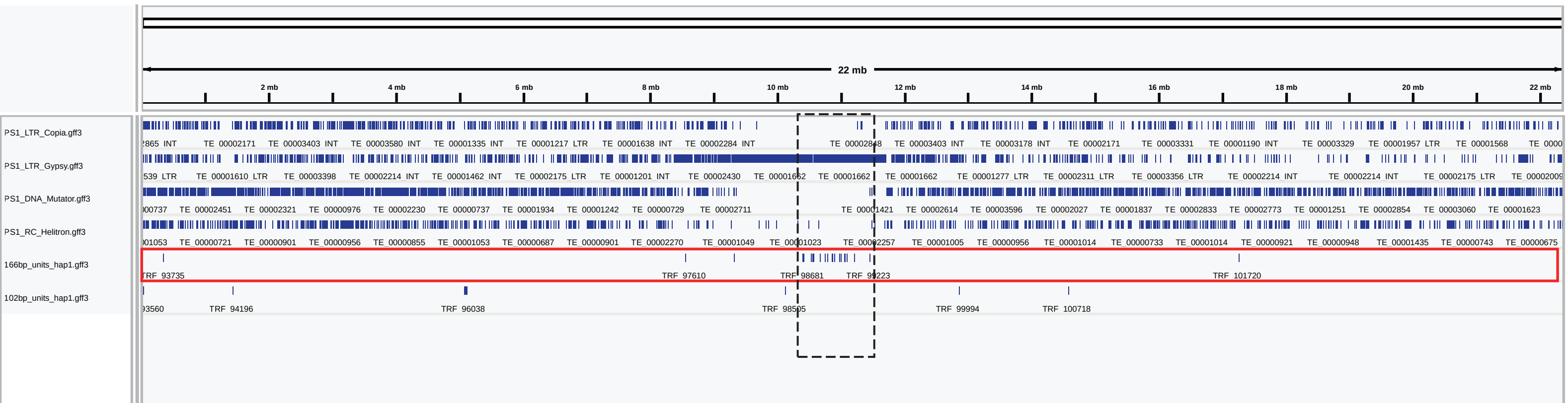

PS_T2T_hap1_chr06

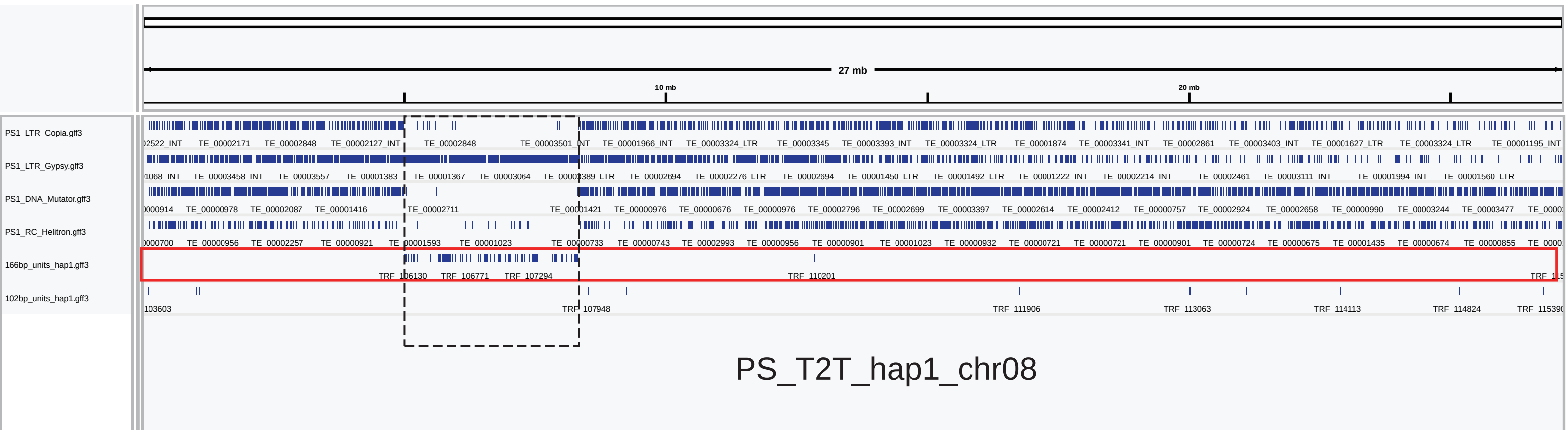
27 mb
10 mb
20 mb
PS1_LTR_Copia.gff3
PS1_LTR_Gypsy.gff3
PS1_DNA_Mutator.gff3
PS1_RC_Helitron.gff3
166bp_units_hap1.gff3
102bp_units_hap1.gff3
PS_T2T_hap1_chr08

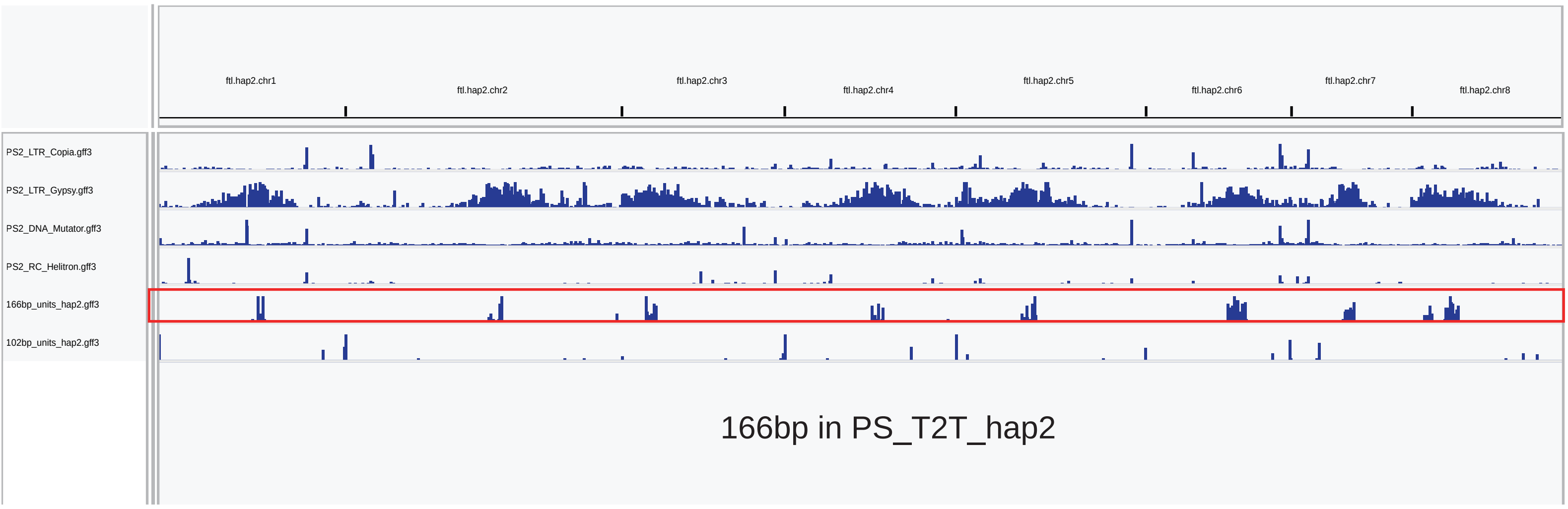
ftl.hap2.chr1
ftl.hap2.chr2
ftl.hap2.chr3
ftl.hap2.chr4
ftl.hap2.chr5
ftl.hap2.chr6
ftl.hap2.chr7
ftl.hap2.chr8
PS2_LTR_Copia.gff3
PS2_LTR_Gypsy.gff3
PS2_DNA_Mutator.gff3
PS2_RC_Helitron.gff3
166bp_units_hap2.gff3
102bp_units_hap2.gff3
166bp in PS_T2T_hap2

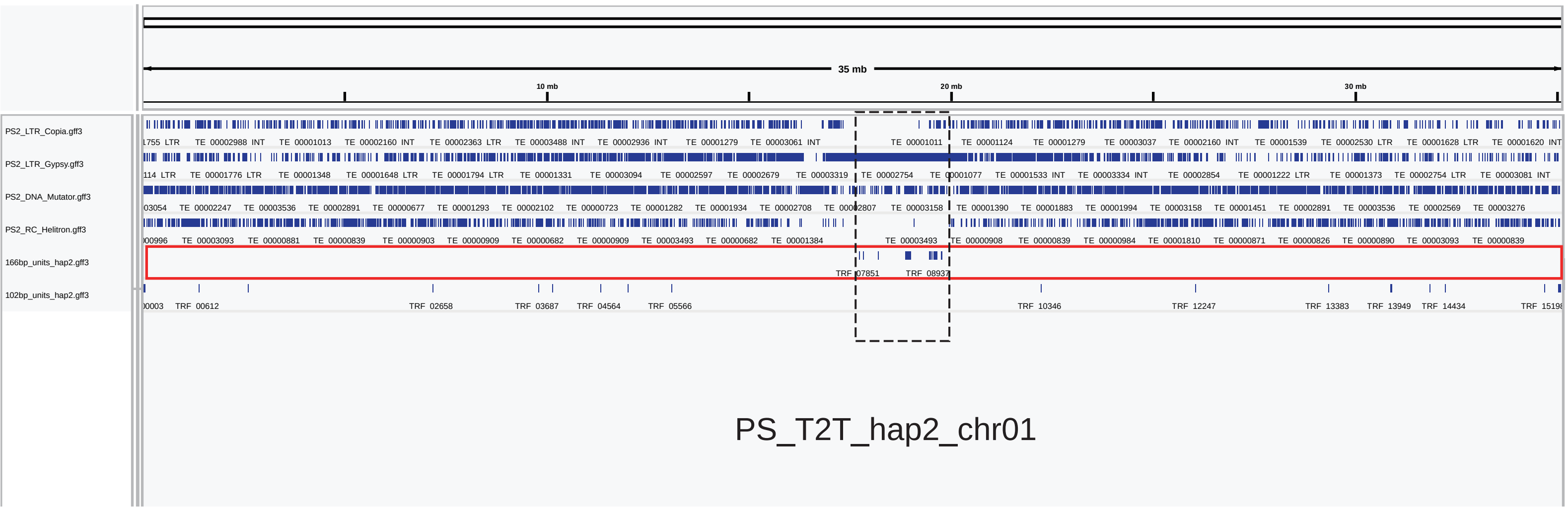
35 mb
10 mb
20 mb
30 mb
PS2_LTR_Copia.gff3
PS2_LTR_Gypsy.gff3
PS2_DNA_Mutator.gff3
PS2_RC_Helitron.gff3
166bp_units_hap2.gff3
102bp_units_hap2.gff3
PS_T2T_hap2_chr01

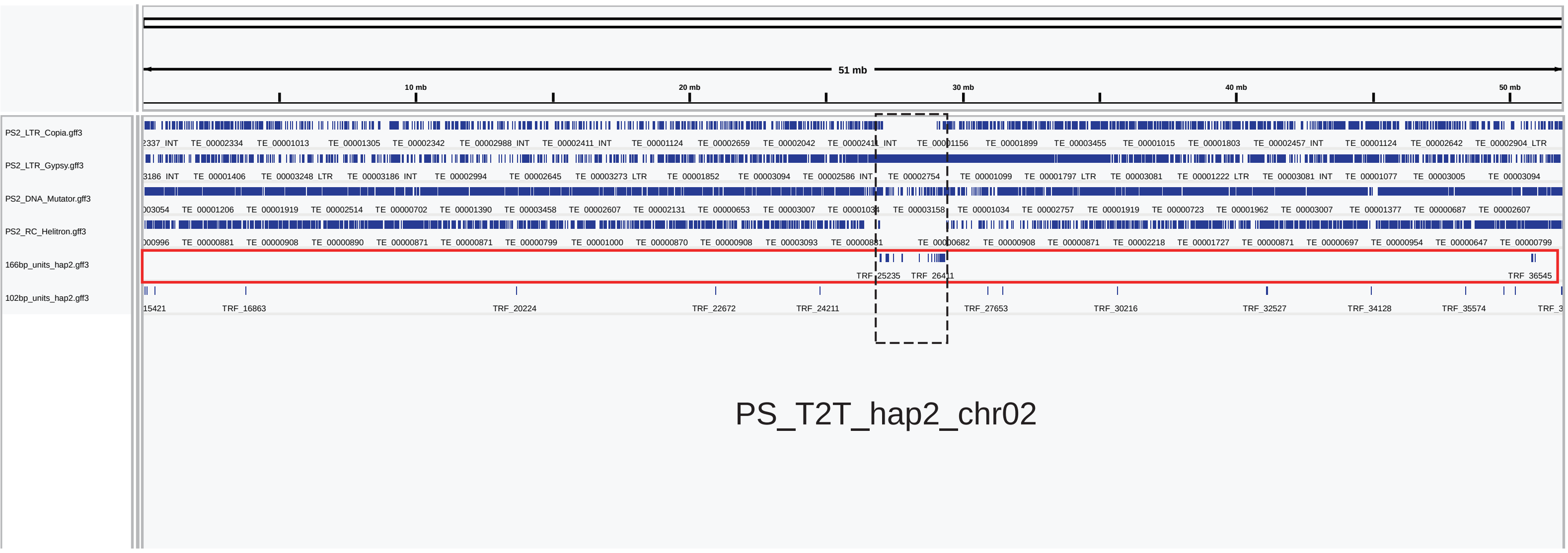
51 mb
10 mb
20 mb
30 mb
40 mb
50 mb
PS2_LTR_Copia.gff3
PS2_LTR_Gypsy.gff3
PS2_DNA_Mutator.gff3
PS2_RC_Helitron.gff3
166bp_units_hap2.gff3
102bp_units_hap2.gff3
PS_T2T_hap2_chr02

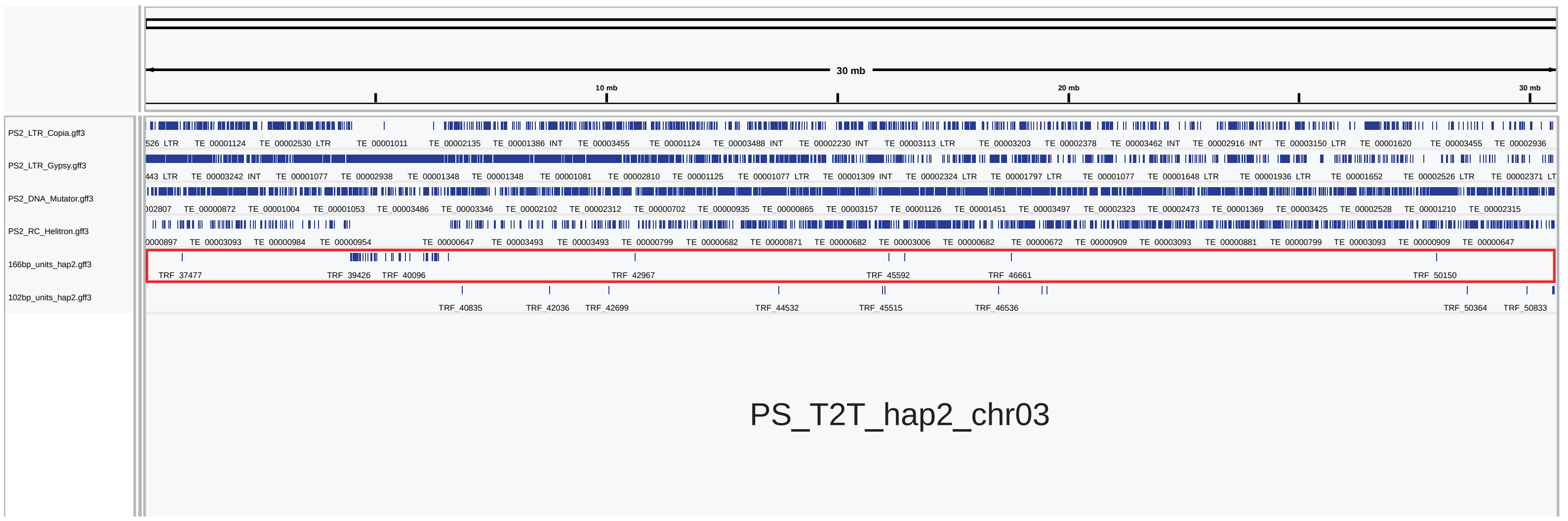
30 mb
10 mb
20 mb
30 mb
PS2_LTR_Copia.gff3
PS2_LTR_Gypsy.gff3
PS2_DNA_Mutator.gff3
PS2_RC_Helitron.gff3
166bp_units_hap2.gff3
102bp_units_hap2.gff3
PS_T2T_hap2_chr03

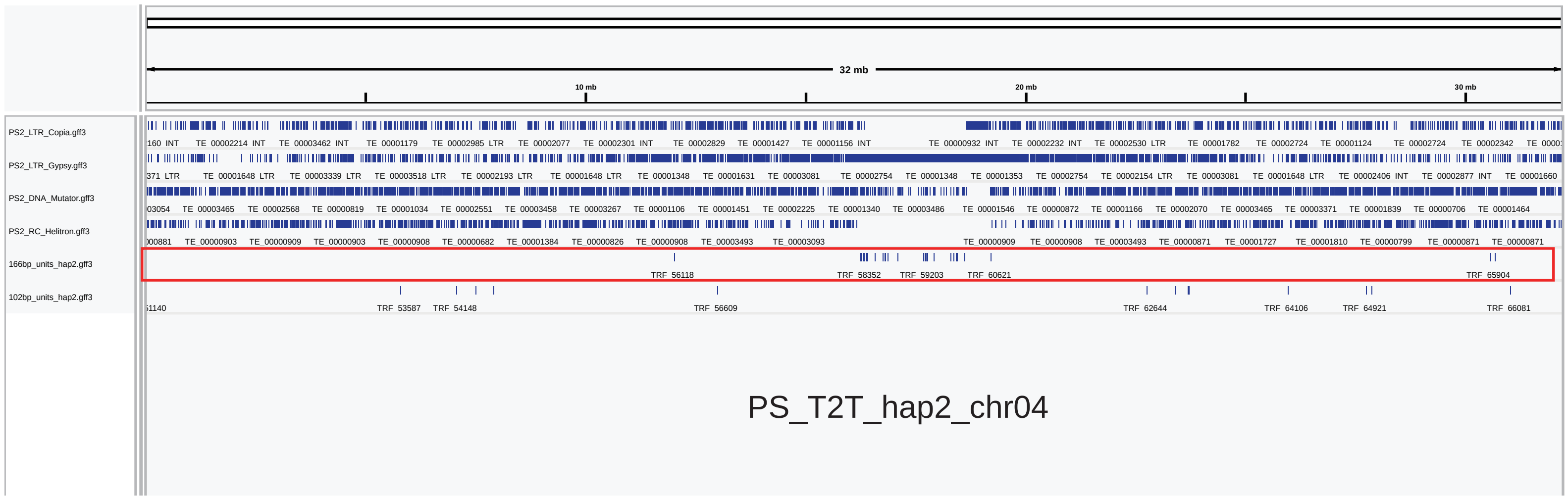
32 mb
10 mb
20 mb
30 mb
PS2_LTR_Copia.gff3
PS2_LTR_Gypsy.gff3
PS2_DNA_Mutator.gff3
PS2_RC_Helitron.gff3
166bp_units_hap2.gff3
102bp_units_hap2.gff3
PS_T2T_hap2_chr04

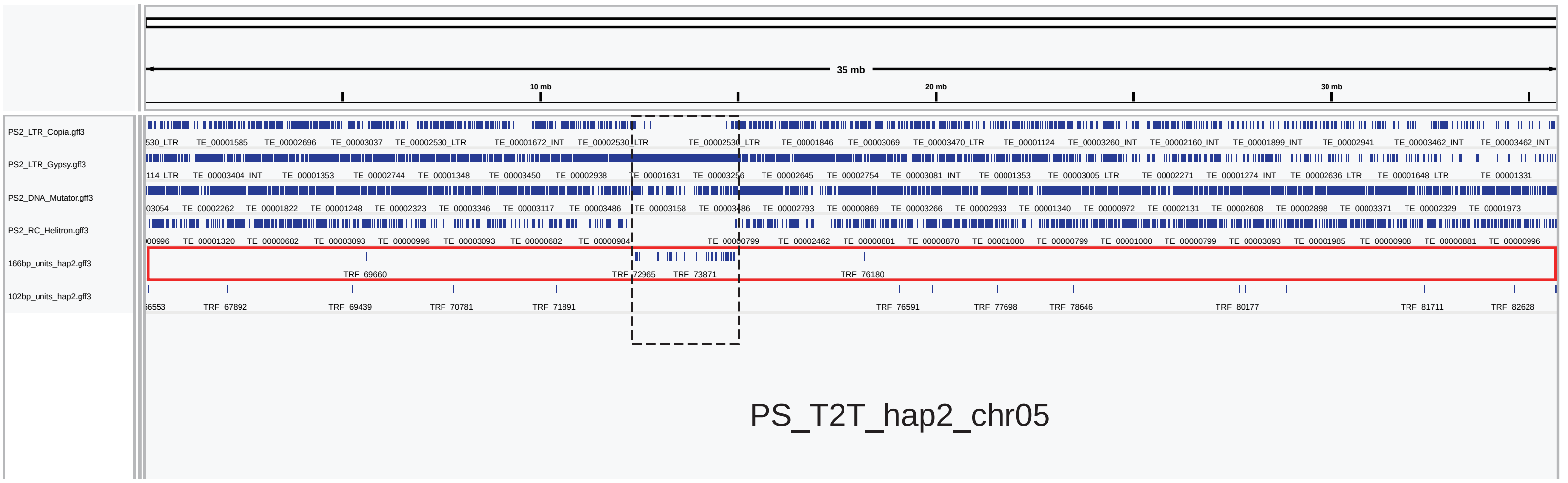
35 mb
10 mb
20 mb
30 mb
PS2_LTR_Copia.gff3
PS2_LTR_Gypsy.gff3
PS2_DNA_Mutator.gff3
PS2_RC_Helitron.gff3
166bp_units_hap2.gff3
102bp_units_hap2.gff3
PS_T2T_hap2_chr05

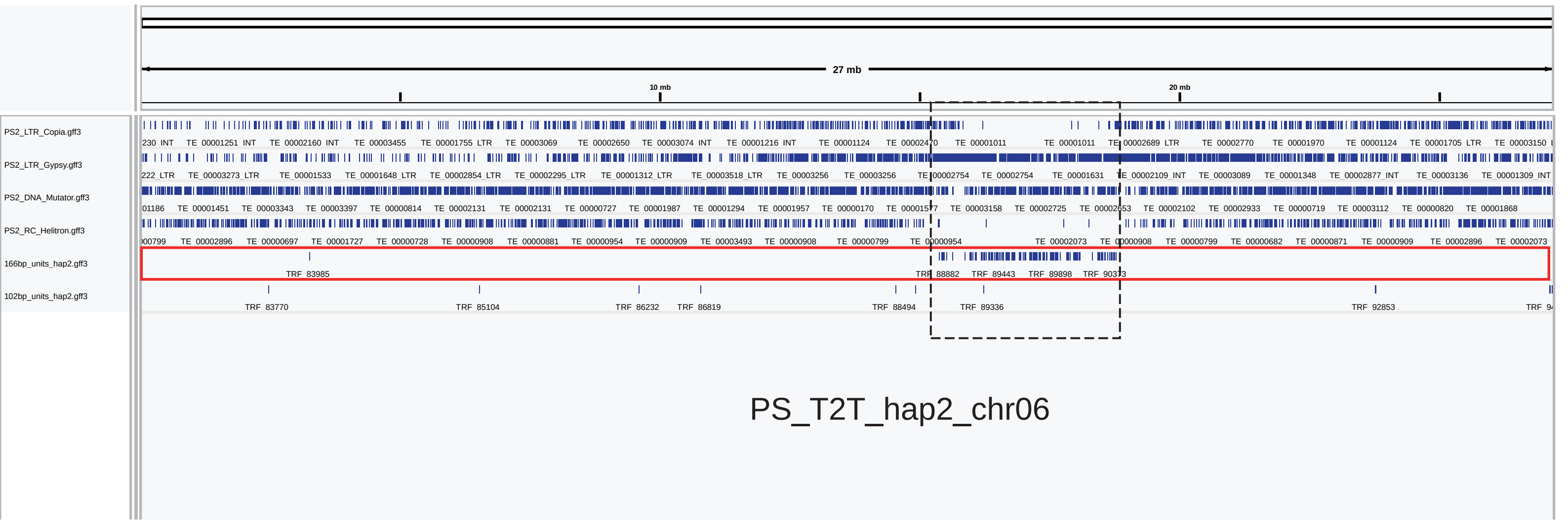
27 mb
10 mb
20 mb
PS2_LTR_Copia.gff3
PS2_LTR_Gypsy.gff3
PS2_DNA_Mutator.gff3
PS2_RC_Helitron.gff3
166bp_units_hap2.gff3
102bp_units_hap2.gff3
PS_T2T_hap2_chr06

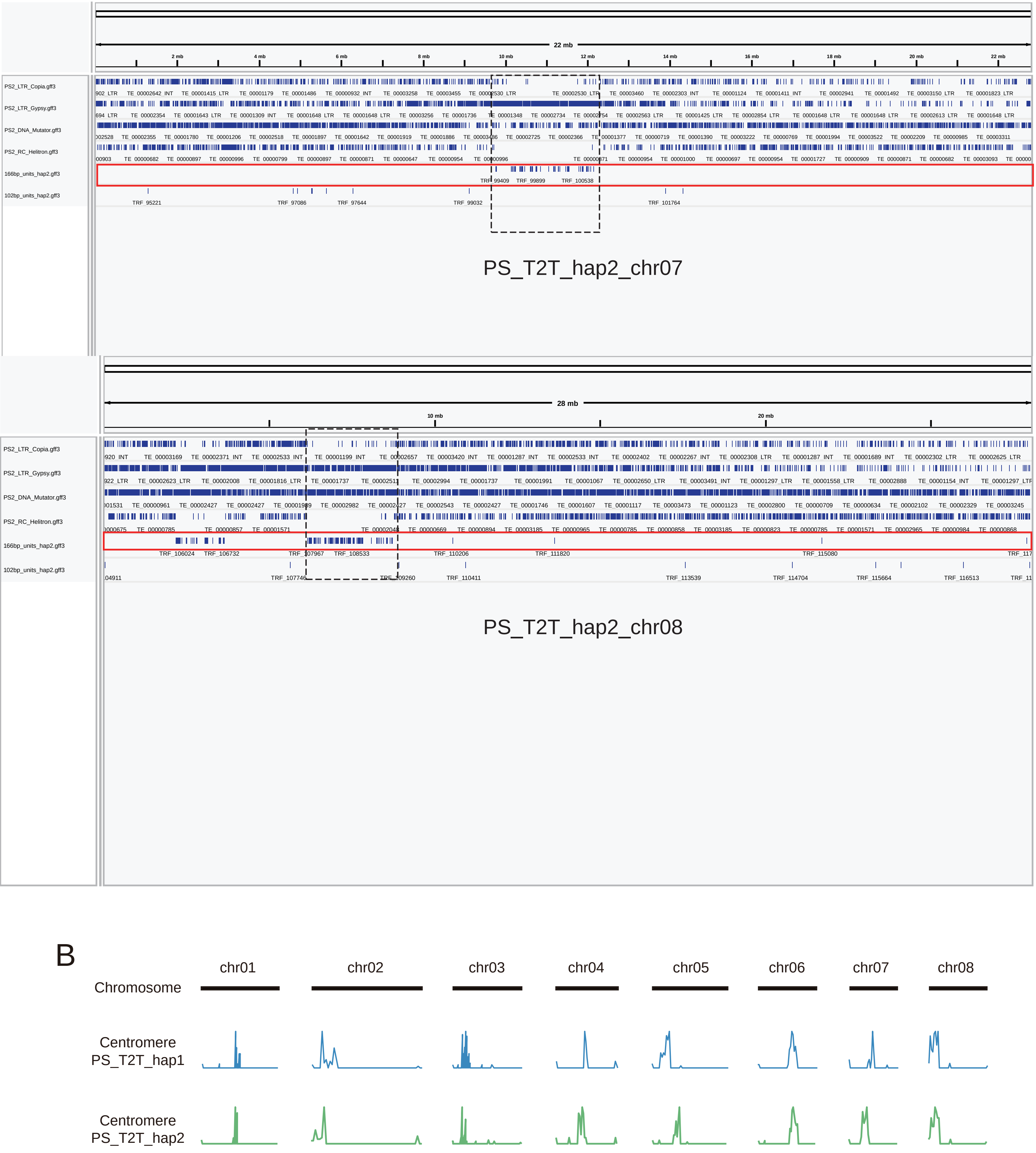

Figure S2 Demonstration of centromeres. (A) Visualization of transposable elements (TEs) sequence and different repeat units on each chromosome in IGV. (B) Dataflow of centromeres prediction (all 166 bp units).

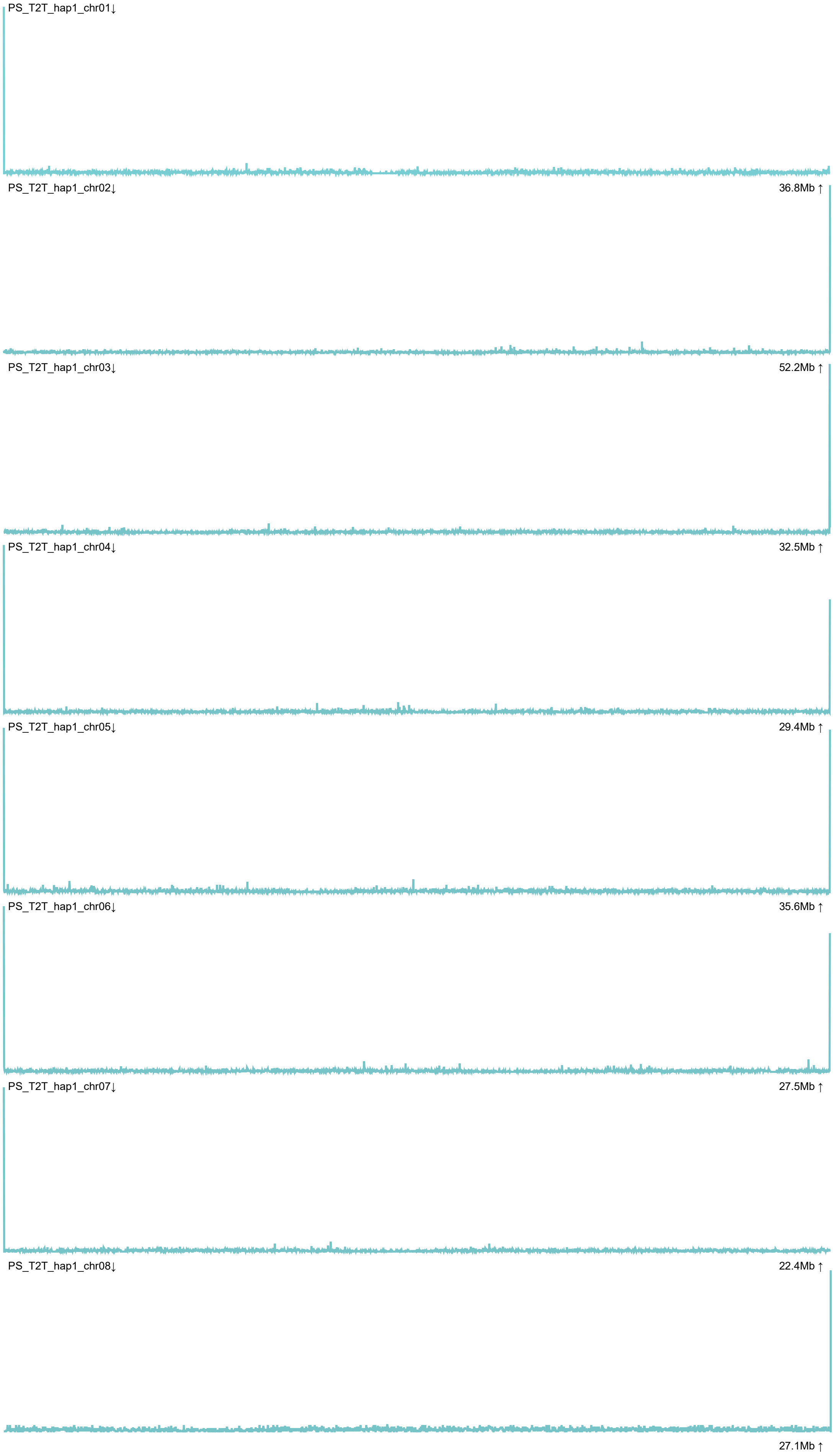

PS_T2T_hap1_chr01↓
PS_T2T_hap1_chr02↓
36.8Mb ↑
PS_T2T_hap1_chr03↓
52.2Mb ↑
PS_T2T_hap1_chr04↓
32.5Mb ↑
PS_T2T_hap1_chr05↓
29.4Mb ↑
PS_T2T_hap1_chr06↓
35.6Mb ↑
PS_T2T_hap1_chr07↓
27.5Mb ↑
PS_T2T_hap1_chr08↓
22.4Mb ↑
27.1Mb ↑

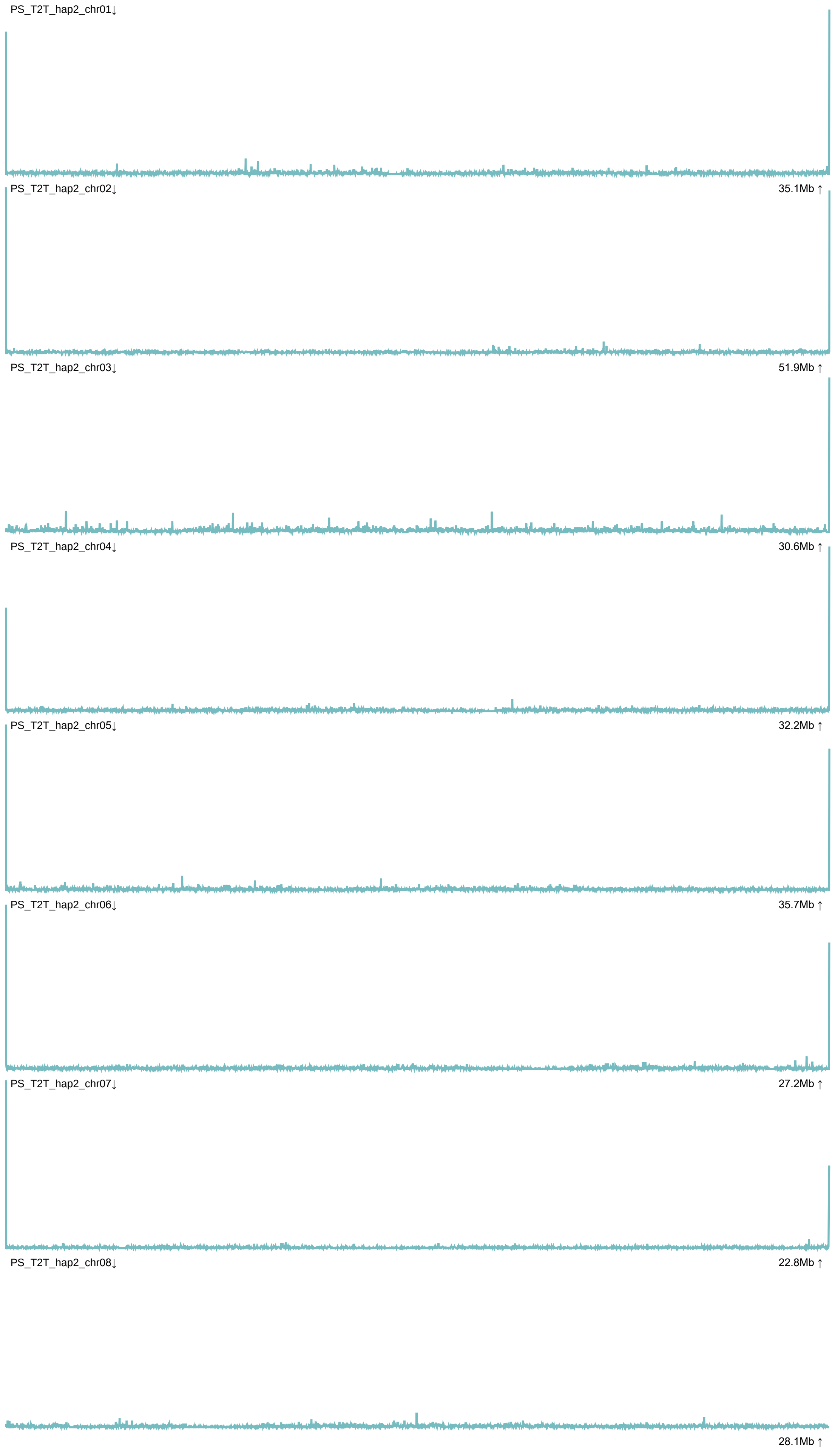

Figure S3 Demonstration of telomeres.

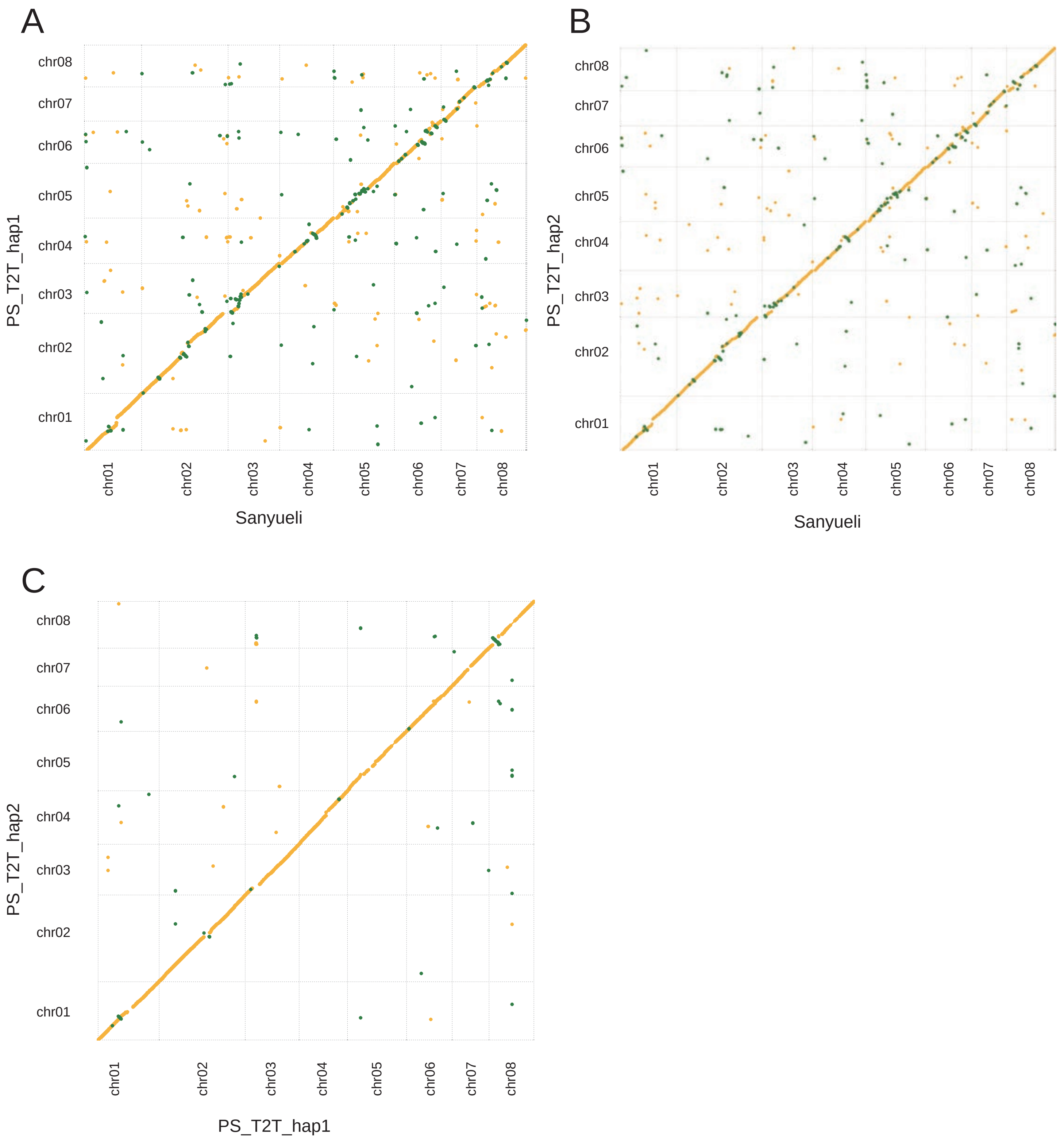

Figure S4 A comparison of genomic collinearity between two haplotypes of PS_T2T and 'Sanyueli' was compared, respectively. (A) PS_T2T_hap1 and 'Sanyueli'. (B) PS_T2T_hap2 and 'Sanyueli'. (C) PS_T2T_hap1 and PS_T2T_hap2. The dotplot illustrates reverse alignments (green).

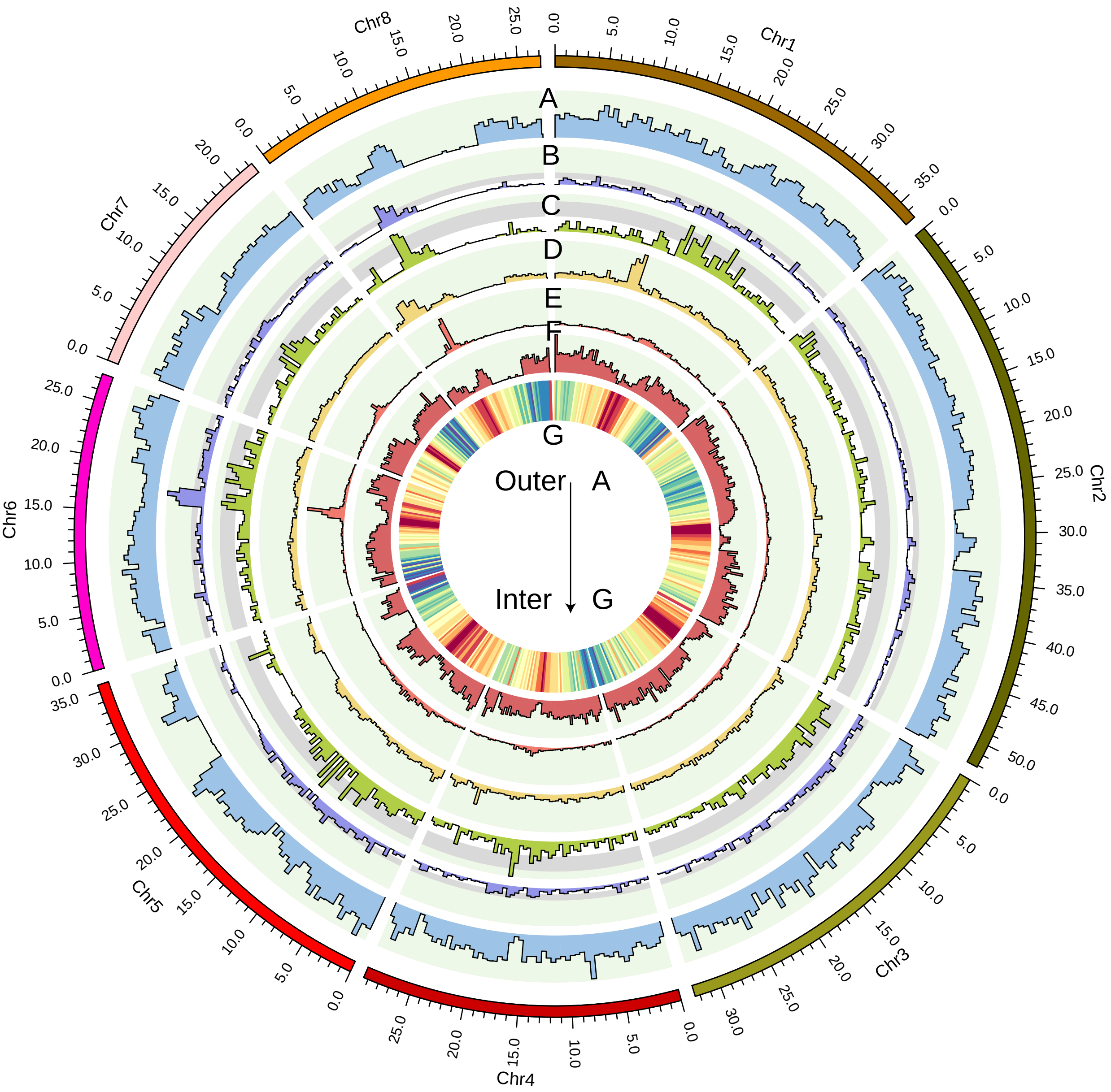

Figure S5 Circos plot of the multidimensional topography of the diploid PS_T2T genome. (A–H), Concentric circles from outermost to innermost, show SNP density (A) Trans. (B) INV. (C) INS. (D) DUP. (E) DEL. (F) Gene density in PS_T2T_hap1.

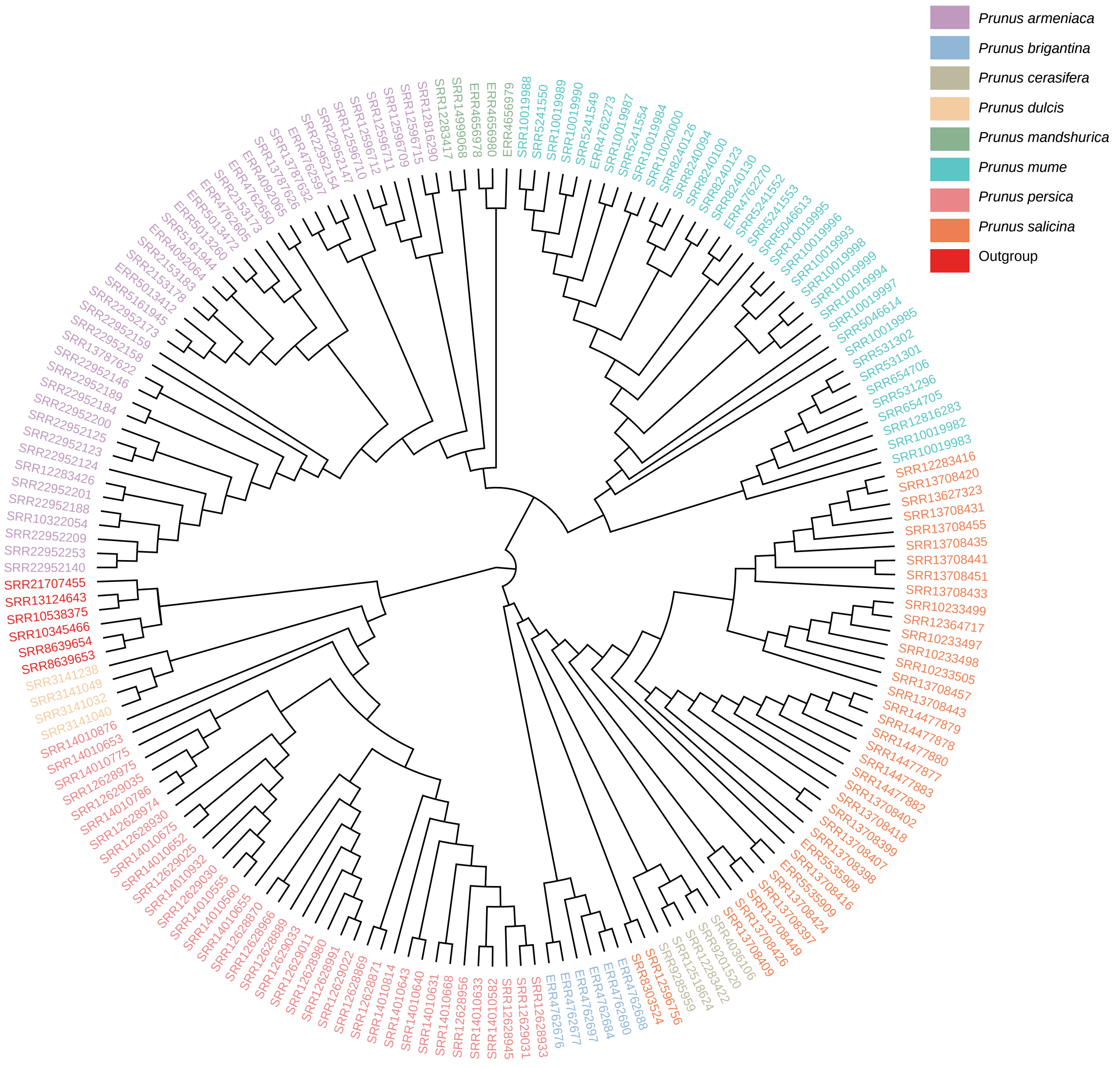

Figure S6 A phylogenetic tree generated based on SNPs using fasttree. The fonts in different colors indicate different group.

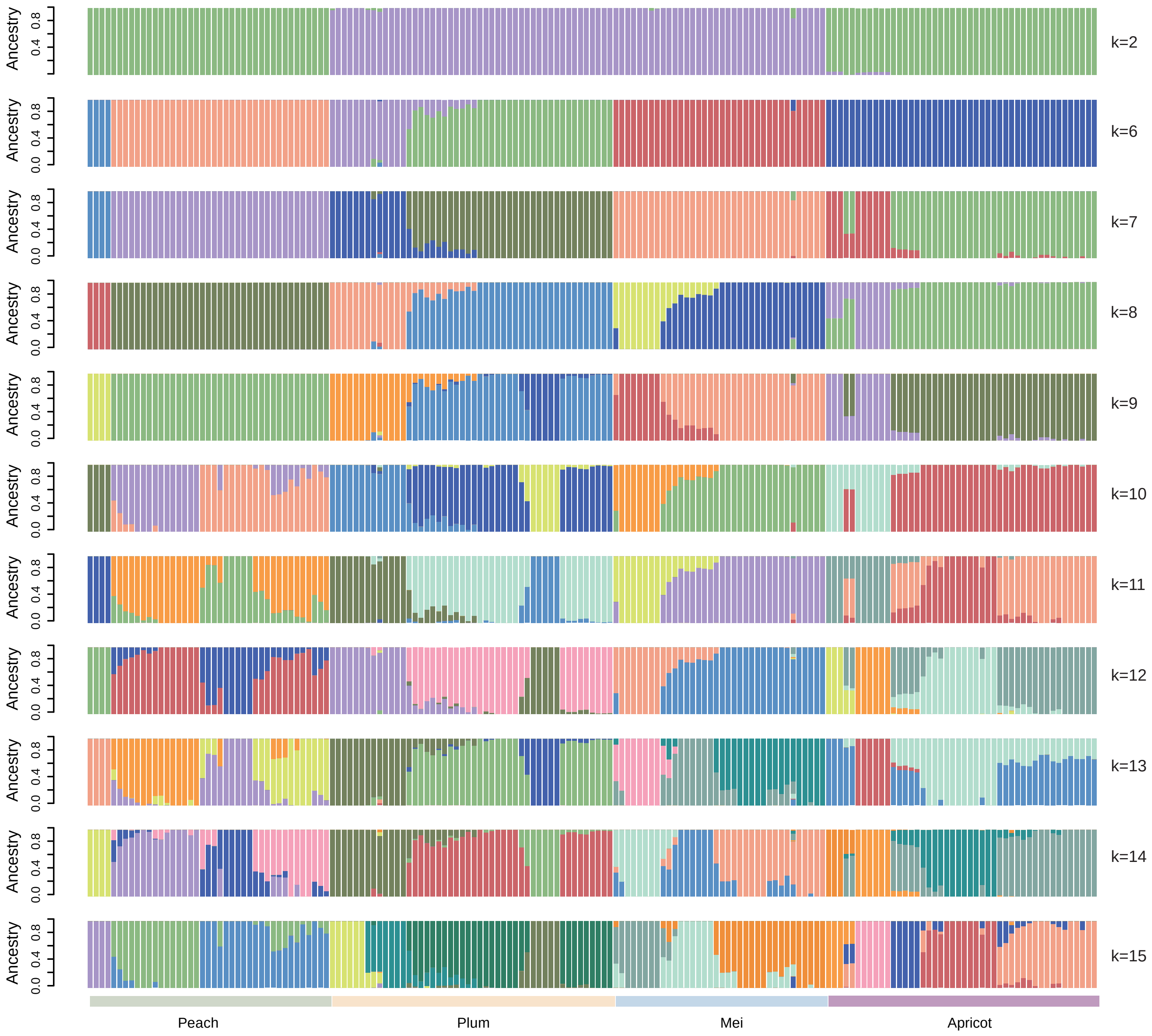

Figure S7 A maximum likelihood phylogenetic tree of 171 accessions in *Prunus* and their population structure determined using ADMIXTURE. The results of admixture analysis from K = 2 to K = 15.

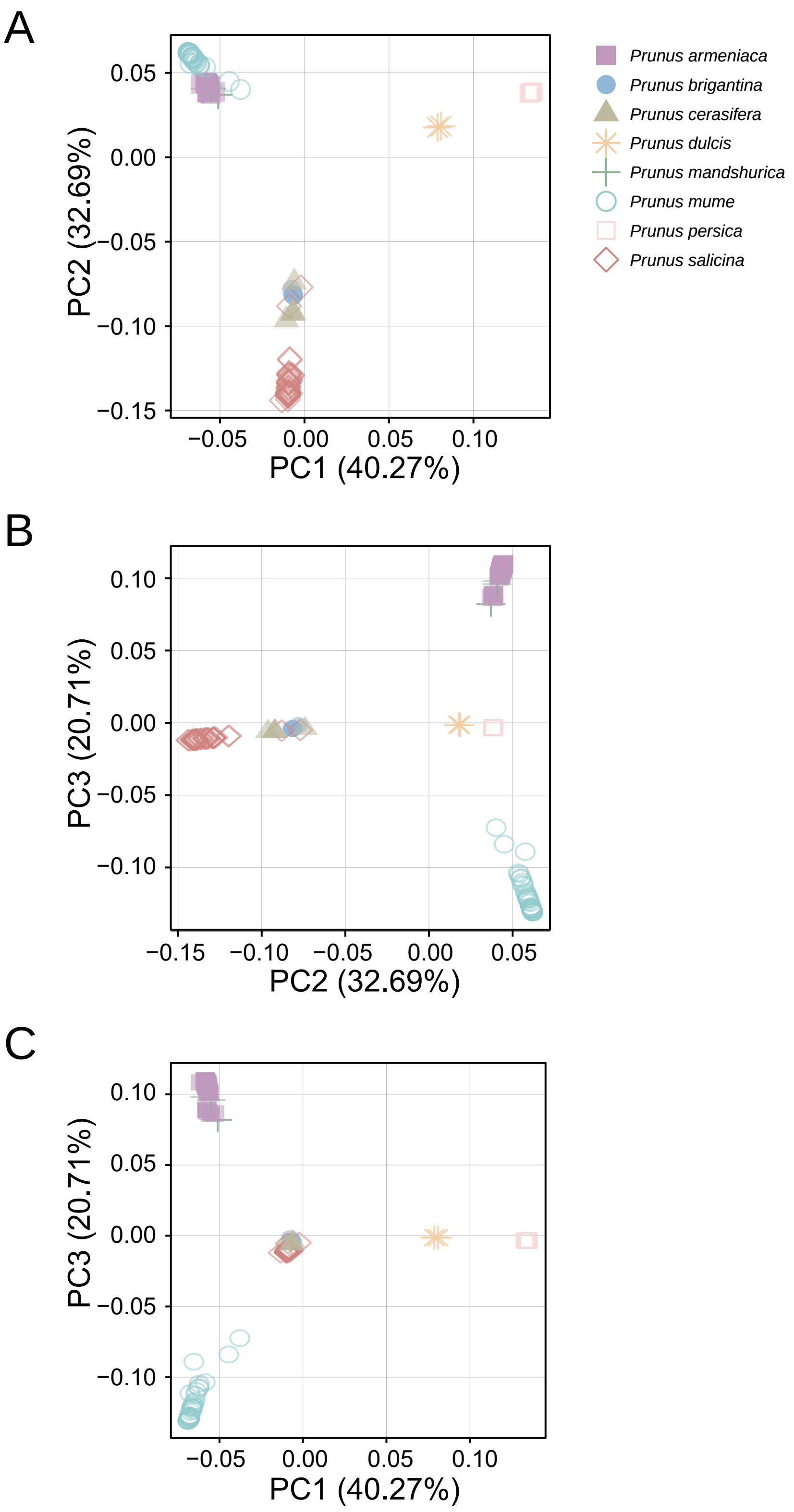

Figure S8 PCA of *Prunus* populations. The different colors suggest different groups.

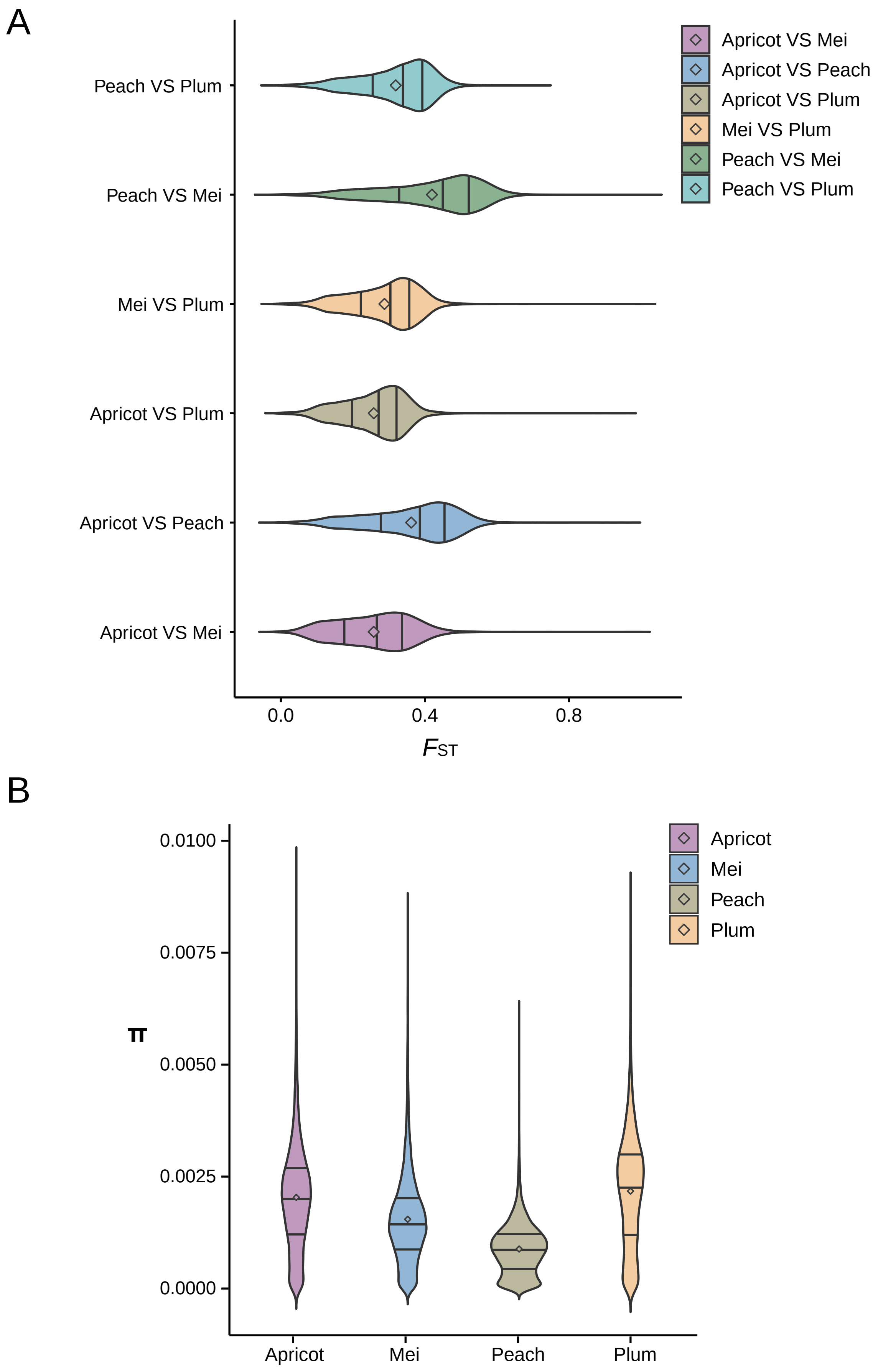

Figure S9 F_{ST} and π between four species. (A) Violin plots indicate the density distribution of F_{ST} in 20 kb window between each group. The dashed lines in each violin plot represent the value of the third quartile, median, and first quartile from right to left, separately. (B) Nucleotide diversity (π) of different groups. Violin plots indicate the density distribution of π in 20 kb window in each group. The dashed lines in each violin plot represent the value of the third quartile, median, and first quartile from right to left, separately.

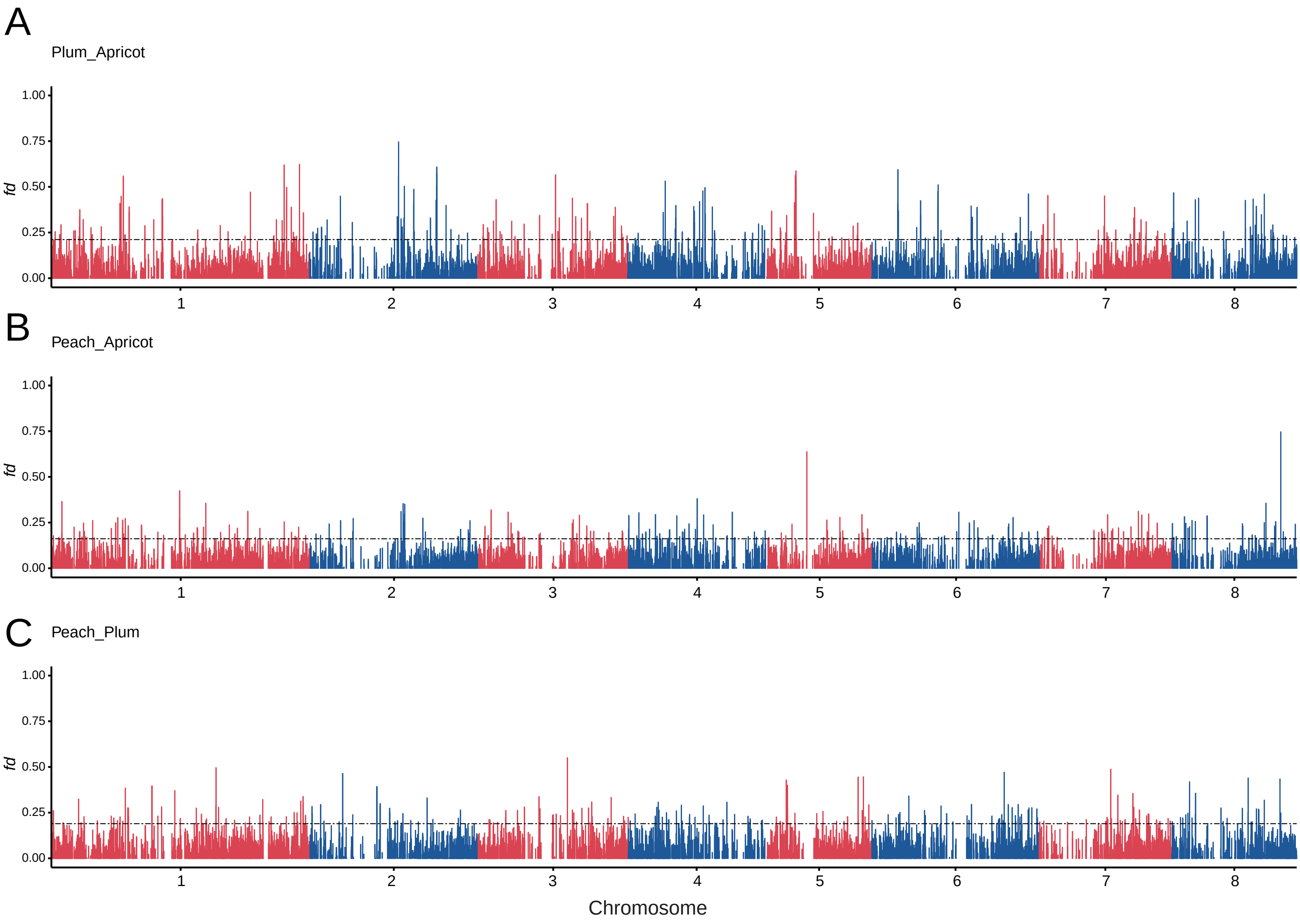

Figure S10 Analysis of the *fd* statistic for estimation of genomewide introgression. We divide the window by 20 kb for calculation. (A) The tested topology is (((Mei, Apricot), Plum), Outgroup). (B) The tested topology is (((Plum, Apricot), Peach), Outgroup). (C) The tested topology is (((Apricot, Plum), Peach), Outgroup).

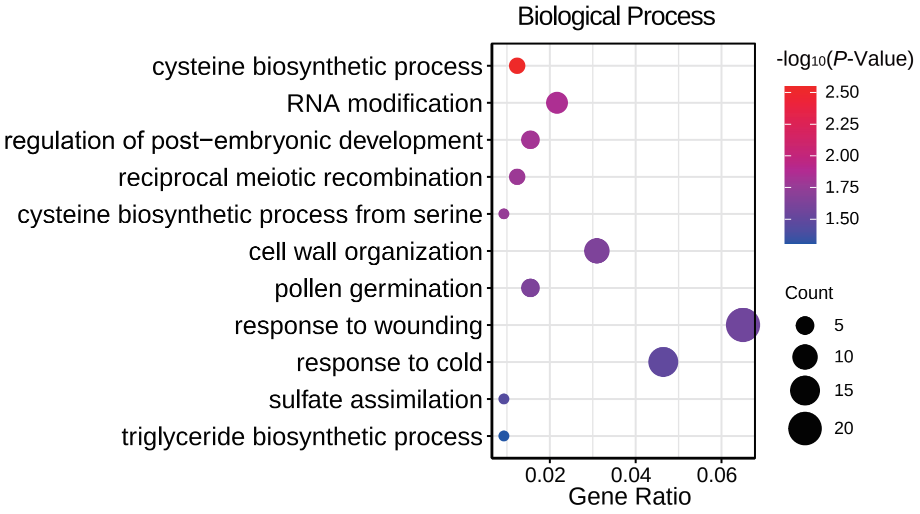

Figure S11 The dot plot shows the GO enrichment of genes located in the top 5% *fd* regions between plum and apricot. The color of the points represents the Benjamini-Hochberg corrected -$\log_{10}$ (*P*-value), and the size of the points represents the number of genes. Gene ratio is the ratio of the number of interested genes annotated in this GO term to all genes.

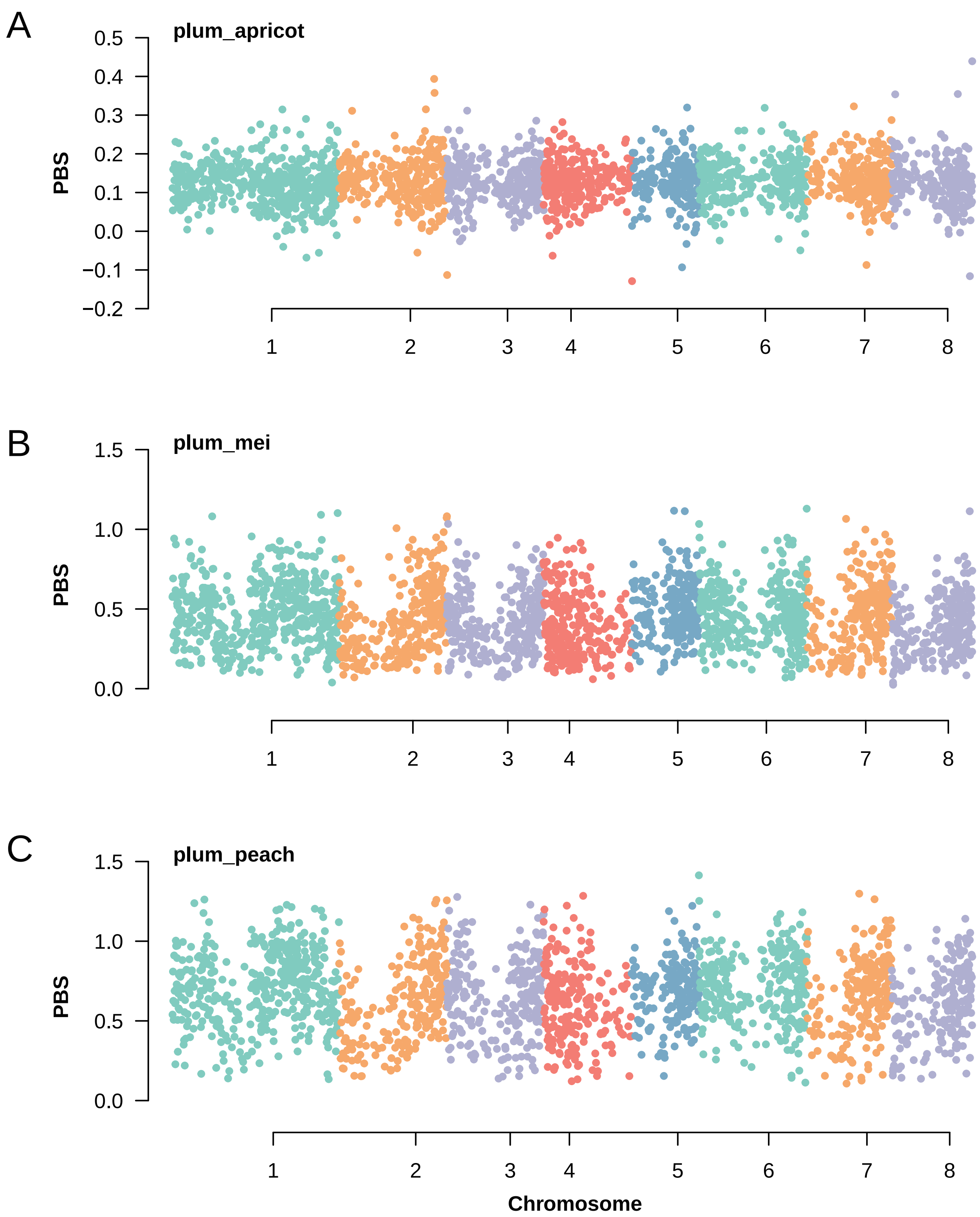

Figure S12. The population branch statistic (PBS) of plum with the other species. PBS was calculated by comparing it with the outgroup.

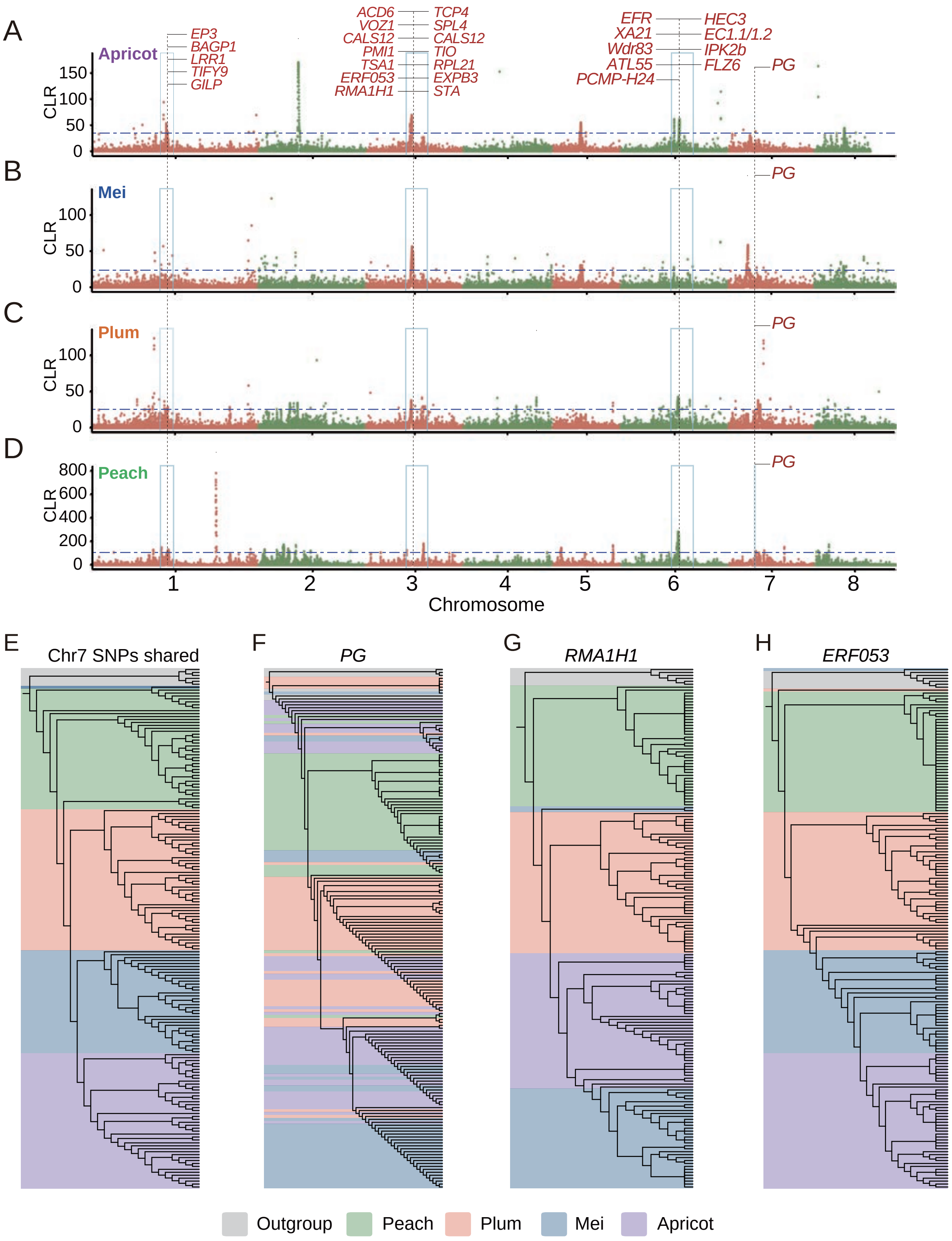

Figure S13 Convergent signals of selection in the APPM complex. (A-D) CLR scores were calculated by SweeD across the genome in apricot, mei, plum, and peach. The dashed lines mark the regions at the top 1%. The red italicized text indicates functional genes common to all four species. (E - H) Evolutionary trees were constructed based on convergence signals within the region, with a particular focus on the SNPs located on chromosome 7 (E), SNP markers in *PG* genes on chromosome 7 (F), and Chromosome 3 (RMA1H1, *ERF053*).

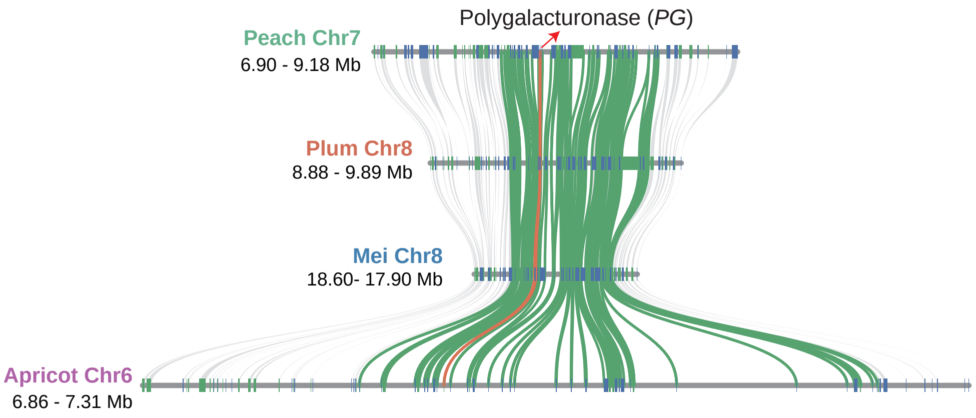

Figure S14 Comparative genome analysis of peach reveals a shared region of 2.02 Mb (Chr7: 6.91 - 8.93 Mb) with the other three species. Green indicates co-linearity across the four genomes. Red syntenic blocks highlight PG loci associated with agronomic traits.

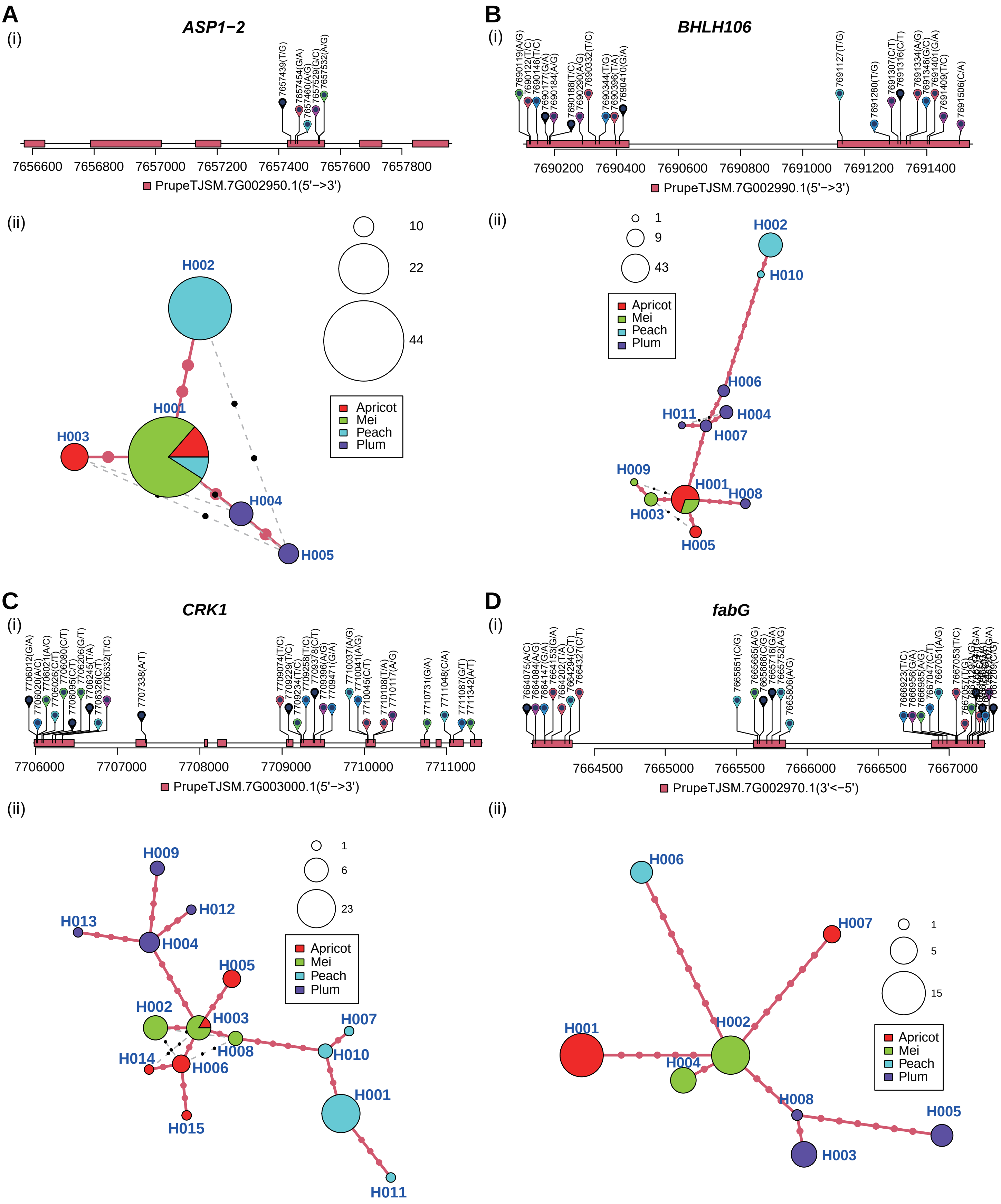
A
ASP1−2
(i)
7657439(T/G)
7657454(G/A)
7657460(A/G)
7657529(G/C)
7657532(A/G)
7656600 7656800 7657000 7657200 7657400 7657600 7657800
PrupeTJSM.7G002950.1(5'−>3')
(ii)
10
22
44
Apricot
Mei
Peach
Plum
H001
H002
H003
H004
H005
B
BHLH106
(i)
7690119(A/G)
7690122(T/C)
7690146(T/C)
7690177(G/A)
7690184(A/G)
7690188(T/C)
7690290(A/G)
7690332(T/C)
7690344(T/G)
7690396(T/A)
7690410(G/A)
7691127(T/G)
7691280(T/G)
7691307(C/T)
7691316(C/T)
7691334(A/G)
7691346(G/C)
7691401(G/A)
7691409(T/C)
7691506(C/A)
7690200 7690400 7690600 7690800 7691000 7691200 7691400
PrupeTJSM.7G002990.1(5'−>3')
(ii)
1
9
43
Apricot
Mei
Peach
Plum
H001
H002
H003
H004
H005
H006
H007
H008
H009
H010
H011
C
CRK1
(i)
7706012(G/A)
7706020(A/C)
7706021(A/C)
7706026(C/T)
7706080(C/T)
7706095(C/T)
7706206(G/T)
7706245(T/A)
7706326(C/T)
7706332(T/C)
7707338(A/T)
7709074(T/C)
7709229(T/C)
7709234(T/C)
7709258(T/C)
7709378(C/T)
7709396(A/G)
7709471(G/A)
7710037(A/G)
7710041(A/G)
7710045(C/T)
7710108(T/A)
7710117(A/G)
7710731(G/A)
7711048(C/A)
7711087(G/T)
7711342(A/T)
7706000 7707000 7708000 7709000 7710000 7711000
PrupeTJSM.7G003000.1(5'−>3')
(ii)
1
6
23
Apricot
Mei
Peach
Plum
H001
H002
H003
H004
H005
H006
H007
H008
H009
H010
H011
H012
H013
H014
H015
D
fabG
(i)
7664075(A/C)
7664084(A/G)
7664147(G/A)
7664153(G/A)
7664294(C/T)
7664327(C/T)
7665651(C/G)
7665665(A/G)
7665666(C/G)
7665716(G/A)
7665752(A/G)
7665806(A/G)
7666923(T/C)
7667051(A/G)
7667053(T/C)
7664500 7665000 7665500 7666000 7666500 7667000
PrupeTJSM.7G002970.1(3'<−5')
(ii)
1
5
15
Apricot
Mei
Peach
Plum
H001
H002
H003
H004
H005
H006
H007
H008

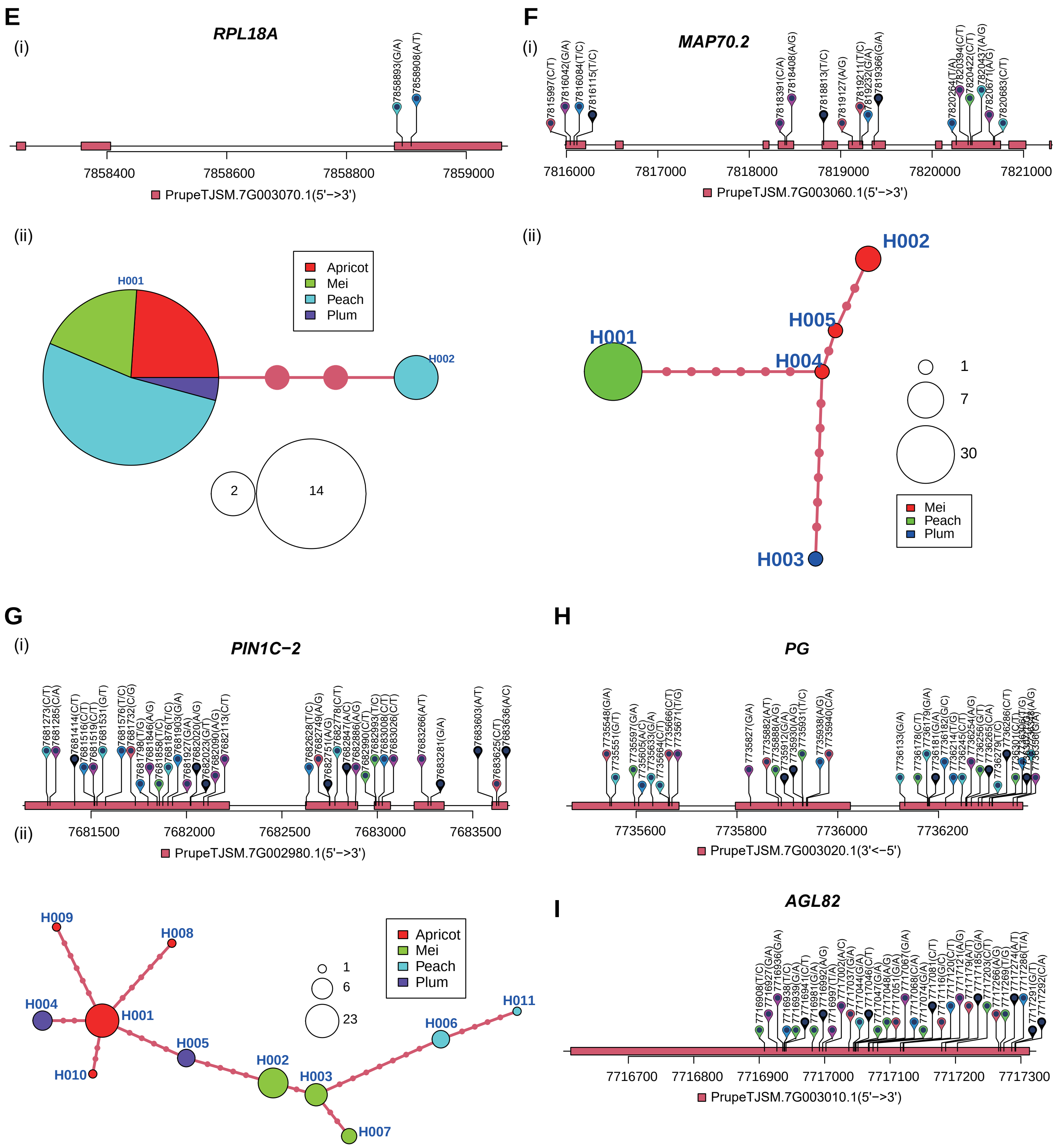

Figure S15 Haplotype analysis of convergent signals of selection is conducted using SNP markers in coding regions (CDS) of relevant genes on chromosome 7 within the APPM complex. The information for each locus is as follows: (i) Visualization of variant positions above the gene model: the black line represents the genome, and rectangles represent exons. (ii) Proportion of different groups within the APPM complex among haplotypes and haplotype network analysis. Numbers above the lines between haplotypes indicate the number of SNPs between them. Percentages indicate the proportion of each group within the APPM complex carrying that haplotype.

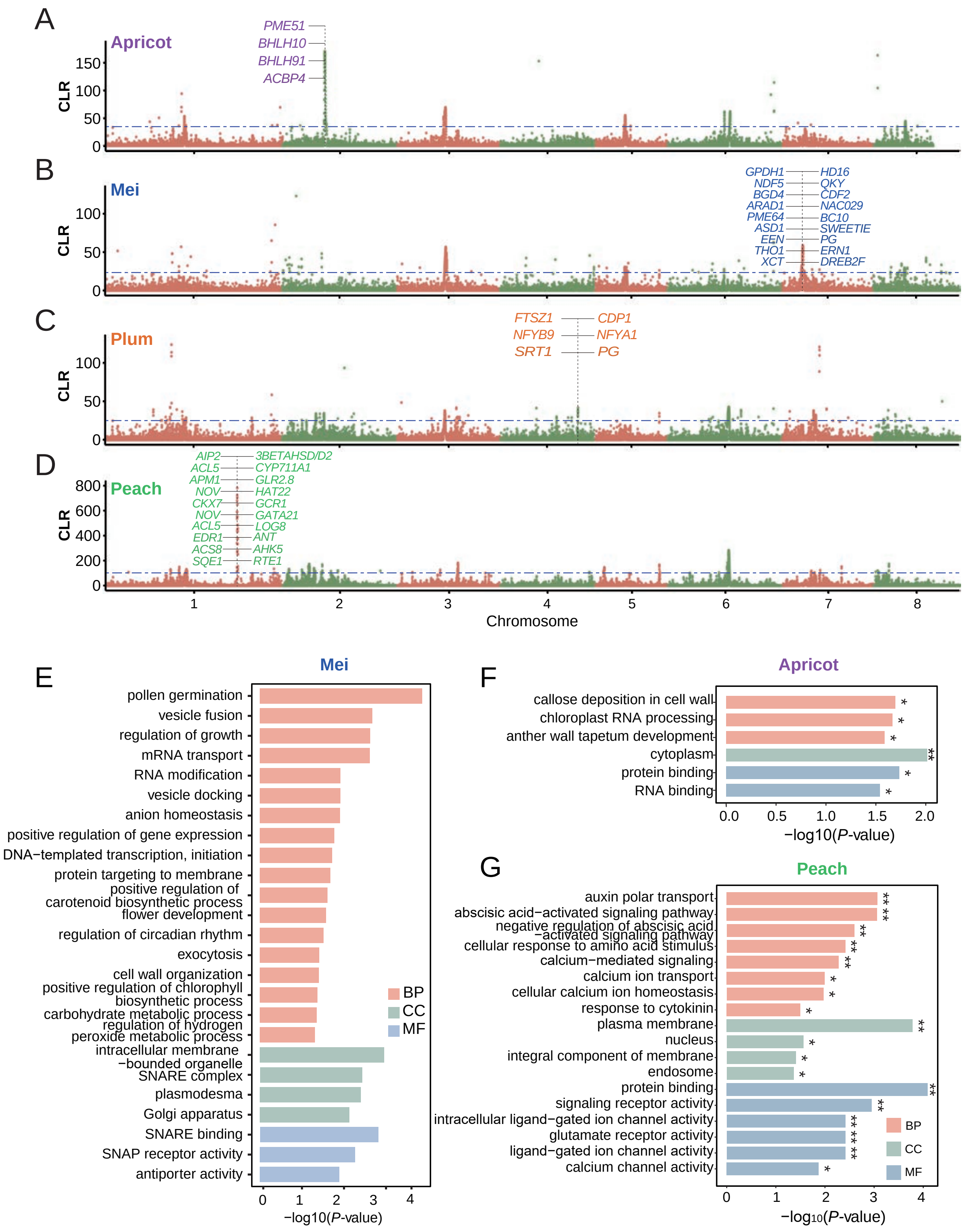

Figure S16 Divergent signals of selection in the APPM complex. (A-D) CLR scores were calculated by SweeD across the genome in apricot, mei, plum, and peach. The dashed lines mark the regions at the top 1%. The purple, blue, orange, and green italicized text represent functional genes unique to apricot, mei, plum, and peach, respectively. (E) GO enrichment analysis of private genes shown on chromosome 7 in mei. The enriched GO term for unique genes in apricot (F) and peach (G). Different colors represent different categories. MF, molecular function; CC, cellular component; BP , biological process. Significant P-values for enrichment: **P*-value < 0.05; ***P*-value < 0.01.
