# Supplementary material for: Comparative population genomics reveals convergent and divergent selection in the apricot–peach–plum–mei complex
Source: Hortic Res. 2024 Apr 16;11(6):uhae109. doi: 10.1093/hr/uhae109 (PMC11179850; doi:10.1093/hr/uhae109)
Supplement: Web_Material_uhae109 [file web_material_uhae109.zip › Supplementary_Materials and methods_HR-2023-843_uhae109.pdf]

Materials and methods

Plant materials, DNA extraction, and sequencing

'Fengtangli' is a high-quality plum variety officially named in 2016 and awarded the National Geographical Indication in 2017. The fruit is nearly round, with light yellow waxy surface, containing 16.1% soluble solids and 13.54% soluble sugar. Plant materials were collected from HuiShui County, Guizhou Province. After harvesting, the samples were promptly frozen in liquid nitrogen and stored at -80°C for DNA extraction. The genomic DNA was extracted from leaf tissue samples using the CTAB method. The concentration and purity of the DNA were assessed with a Qubit fluorometer, and DNA integrity was verified through gel electrophoresis. For Hi-C library construction, isolated nuclei were lysed with 0.1% SDS at 65°C for 10 minutes and quenched with 1% Triton X-100. DNA in the nuclei was digested with MboI at 37°C for 2 hours, and fragment ends were labeled with biotinylated cytosine nucleotides. Blunt-end ligation was performed at 16°C overnight using T4 DNA ligase. After ligation, cross-linking was reversed by proteinase K at 65°C overnight. DNA purification was carried out using the QIAamp DNA Mini Kit, and the purified DNA was sheared to approximately 400 base pairs. Point ligation junctions were pulled down using Dynabeads® MyOne™ Streptavidin C1. The Hi-C library for Illumina sequencing was prepared with the NEBNext® Ultra™ II DNA Library Prep Kit for Illumina. Fragments between 400 and 600 base pairs were paired-end sequenced on the Illumina HiSeq X Ten platform in 150PE mode. Two replicates were generated for each group material, ensuring robust and reliable results for the Hi-C analysis. For HiFi sequencing, single-molecule real-time cells were sequenced on the PacBio Sequel II platform using the Circular Consensus Sequencing (CCS) method with default parameters (https://github.com/PacificBiosciences/ccs). The raw sequencing data underwent preprocessing using the SMRTlink software, which included adapter removal and self-correction of subreads to generate high-quality HiFi sequences for downstream analysis. The SMRTlink parameters were set to ensure data quality, including minimum subread length, maximum subread length, minimum number of sequencing passes, and

minimum predicted accuracy.

Genome De Novo Assembly and Quality Assessment

The HiFi and Hi-C data were assembled using HiFiasm v0.19.5 [1,2] with default parameters to obtain two contig-level haplotype genomes. Genome heterozygosity was estimated using a k-mer-based approach by GenomeScope v2.0 [3]. To improve the assembly, the Hi-C data were utilized to anchor and remove some short contigs. RagTag v2.1.0 [4] was employed to establish the preliminary arrangement of contigs on chromosomes, with 'Sanyueli' serving as the reference genome. Further, we used Juicer v1.5 [5] to anchor the Hi-C sequencing data and subsequently combined it with 3D-DNA v180922 [6] for scaffold-level assembly. The obtained results were manually adjusted using Juicebox [7] before running 3D-DNA again to obtain the genome at the scaffold level. To verify the correctness of gap filling, Minimap2 v2.24-r1122 [8] was used to compare the original HiFi data for sequence comparison. The resulting sequence positions of gaps were located using IGV v2.12.3 [8], and the gaps were subsequently filled in the genome using Minimap2. The genome completeness was evaluated using BUSCOs with the embryophyta_odb10 database [9], while the genome continuity was assessed by calculating the contig N50 length.

Identification of Telomeres and Centromeres

To identify telomeres, we used the telomere pipeline developed by TIDK v.0.2.0 (https://github.com/tolkit/telomeric-identifier), which identifies telomeric sequences characterized by 'CCCATTT' at the 5' end and 'TTTAGGG' at the 3' end. We successfully identified 24 out of the expected 32 telomeres (8 chromosomes of 2 haplotypes). The telomere peak line was visualized using R scripts based on rapid statistics of telomeres. Tandem repeat annotation was performed using TRF v4.09, and the results were merged using TRF2GFF [10]. Data statistics and visualization were completed by extracting information using the awk command in the Linux system and analyzing the results in IGV. The centromeres were then identified by comparing the results with TE annotation and TRF using IGV. To detect centromeric regions, we

scanned candidate repeats from 30 to 500 bp along the genome. Tandem Repeats Finder (TRF) (https://tandem.bu.edu/trf/trf.html) found 470 different repeat units in the PS_T2T assemblies. When we qualify the conditions as copies greater than 10 and periods between 100 bp and 300 bp, the 166 bp repeats were the most abundant unit in the whole genome.

Genome annotation and identified transposable elements

The raw RNA sequence data were subjected to a filtering process to eliminate adaptor sequences using Trimmomatic v.0.39 [11], then low-quality reads with more than 10% ambiguous nucleotides (N), and 50% of bases with a quality value ≤5, utilizing HISAT2 v.2.2.1 [12] and StringTie v.2.1.7 [13]. The clean data were subsequently aligned to the assembly genome using the default parameter. Gene annotation followed the protocol outlined in the Genome-Wide-Annotation-Pipeline (https://github.com/unavailable2374/Genome-Wide-Annotation-Pipeline). For functional gene annotation, Interproscan v5.29 [14] was employed, leveraging blast results from a range of databases, such as Pfam, UniProt, GO, and KEGG.

Comparative genomics and gene families

For whole-genome alignment, Minimap2 was used to align the genomes, and the alignment BAM file was indexed using SAMtools v1.11 [15]. We then deployed the Synteny and Rearrangement Identifier (SyRI) to identify collinear orthologs and structural rearrangements between two haplotypes of the PS_T2T and 'Sanyueli' genome [16]. For a visual interpretation of the data, the Plotsr tool (https://github.com/schneebergerlab/plotsr) was used. Additionally, for an in-depth PS_T2T's genome comparison with the 'Sanyueli' reference genome, we employed MUMmer v4.0 [17]. Initially, we aligned the two genome sequences using nucmer (nucmer --mum) and filtered the results (delta-filter -i 95 -l 10000). Subsequently, we used gnuplot to visualize the comparison results as dot plots. Detailed comparison data were extracted using the 'show-coords -T -q -H' command.

Mcscan toolkit implemented in Python

(https://github.com/tanghaibao/jcvi/wiki/MCscan-[Python-version]) was used to identify structure variations among the apricot, peach, plum, and mei. Briefly, the 'jcvi.compara.catalog' module with parameter 'python -m jcvi.compara.catalog ortholog --no_strip_names A B --cscore=.99 --cpu=1' was executed to detect orthologs; then, 'jcvi.compara.synteny' module with 'python -m jcvi.compara.synteny screen --minspan=30 --simple A.B.anchors A.B.anchors.new' was used to establish the syntenic regions; finally, 'jcvi.graphics.karyotype' module was carried out to visualize the syntenic relationships. Additionally, the microsynteny visualization was performed using the command 'python -m jcvi.graphics.synteny blocks2 peach_plum_mei_apricot.bed blocks2.layout'.

Lastly, to ascertain gene families in apricot, peach, plum, and mei, Orthofinder v2.5.2 [18] was utilized, comparing their protein-coding gene sequences. For enrichment analysis of the species-specific gene sets, we employed Davied (https://david.ncifcrf.gov/tools.jsp). Data visualization was achieved with the ggplot package in R.

Phylogenetic and population structure analyses

We analyzed illumina raw reads from 177 samples sourced from the Short Read Archive (SRA) at NCBI **(Supplementary Data Table S13)**. For SNP calling, we aligned the illumina short reads to the TJSM genome with BWA-MEME, then sorted them with SAMtools [15]; PCR duplicates were removed using Genome Analysis Toolkit (GATK) [19]. SNPs were identified using GTX [20,21] and subsequently filtered using VCFtools [22] with the following parameters: --mac 4 -minQ 30 -minDP 4 -maxDP 200 -remove-indels. Finally, 3,749,618 SNPs with read depth < 4 and depth > 200 were left for further analysis. individuals with a MAF ≥ 0.05, missing rate $\leq 20\%$ using PLINK v1.9 [23] with the parameters: -geno 0.2 -maf 0.05 was used to build a maximum likelihood phylogenetic tree, as well as to perform population structure and PCA. The phylogenetic tree was built using the FastTree2 [24] program. Population structure was investigated using ADMIXTURE [25] and evaluating each K from 2 to 15 [23]. Population heterozygosity was determined by the formula (N(NM) -

O(HOM))/ N(NM), where N(NM) represents the number of observed variants, and O(HOM) represents the number of observed homozygous variants [26]. Heterozygous sites and π for SNPs in each group were calculated using VCFtools v0.1.15. Fixation indices (F_{ST}) were calculated using the VCFtools with 50-kb nonoverlapping windows. Both the relative divergence measure F_{ST} were estimated to identify domestication and differentiation regions.

Introgression analysis with ABBA-BABA tests and TreeMix

For exploring introgression events and estimating the proportion of introgression, the D, f_d, fdM, and f_b statistics were computed based on the 8,011,997 SNPs dataset. To evaluate introgression probabilities across the genome, Dsuite (https://github.com/millanek/Dsuite) was used to calculate the f4-ratio statistics and Patterson's D (ABBA-BABA) based on the complete SNP dataset and whole-genome phylogeny tree. To evaluate the divergence in introgression region among the groups with introgression signals, we filtered the windows with genotyped variants less than 100 and calculated f_d and fdM using the ABBABABAwindows.py program (https://github.com/simonhmartin/genomics_general). Specifically, we computed D-statistics based on SNP frequency differences. For a triplet of taxa P1, P2, and P3, and an outgroup, that follows the phylogeny of (((P1, P2), P3), Outgroup), a D statistic significantly different from zero indicates P3 exchanged gene with P1 (D value 0) or P2 (D value >0). Furthermore, the population relatedness and migration events were inferred using TreeMix v1.13 (https://speciationgenomics.github.io/Treemix/) [27]. We estimated potential migration events ranging from 1 to 5 and repeated each test 5 times. We then used the plotting_func.R script to highlight and add migration edges and directions to the graph. The PBS method was used to identify positively selected genes in each group. PBS was calculated using PBScan v1.0 (https://github.com/thamala/PBScan), each with 20 kb sliding windows, and windows with the top 5% of values were selected as highly divergent regions.

Detection genome scanning for selective sweep signals.

We employed multiple software tools to identify potential regions under selection within the *Prunus* population. Using SweeD v3.3.2 [28] with default parameters, we calculated the CLR to detect genomic signatures of positive selection in the peach, apricot, plum, and mei, while configuring the 'grid' parameter. The CLR computation was conducted at 1000 grids. The regions with the top 1% CLR were determined as potentially positively selected regions. The genes underlying the outlier windows were then annotated based on the peach annotation.

References

1. Cheng H, Jarvis ED, Fedrigo O et al. Haplotype-resolved assembly of diploid genomes without parental data. *Nat Biotechnol*. 2022;**40**:1332-1335.

2. Cheng H, Concepcion GT, Feng X et al. Haplotype-resolved de novo assembly using phased assembly graphs with hifiasm. *Nat Methods*. 2021;**18**.

3. Ranallo-Benavidez TR, Jaron KS, Schatz MC. GenomeScope 2.0 and Smudgeplot for reference-free profiling of polyploid genomes. *Nat Commun*. 2020;**11**:1432.

4. Alonge M, Lebeigle L, Kirsche M et al. Automated assembly scaffolding using RagTag elevates a new tomato system for high-throughput genome editing. *Genome Biol*. 2022;**23**:258.

5. Durand NC, Shamim MS, Machol I et al. Juicer provides a one-click system for analyzing loop-resolution Hi-C experiments. *Cell Syst*. 2016;**3**:95-98.

6. Dudchenko O, Batra SS, Omer AD et al. De novo assembly of the *Aedes aegypti* genome using Hi-C yields chromosome-length scaffolds. *Science*. 2017;**356**:92-95.

7. Durand NC, Robinson JT, Shamim MS et al. Juicebox provides a visualization system for Hi-C contact maps with unlimited zoom. *Cell Syst*. 2016;**3**:99-101.

8. Thorvaldsdóttir H, Robinson JT, Mesirov JP. Integrative Genomics Viewer (IGV): high-performance genomics data visualization and exploration. *Brief Bioinform*. 2013;**14**:178-192.

9. Seppey M, Manni M, Zdobnov EM. BUSCO: assessing genome assembly and annotation completeness. In: Kollmar M, ed. *Gene Prediction*. Vol 1962. Methods in Molecular Biology. Springer New York; 2019:227-245.

10. Benson G. Tandem repeats finder: a program to analyze DNA sequences. *Nucleic Acids Res*. 1999;**27**:573-580.

11. Bolger AM, Lohse M, Usadel B. Trimmomatic: a flexible trimmer for Illumina sequence data. *Bioinformatics*. 2014;**30**:2114-2120.

12. Kim D, Paggi JM, Park C et al. Graph-based genome alignment and genotyping with HISAT2 and HISAT-genotype. *Nat Biotechnol*. 2019;**37**:907-915.

13. Pertea M, Pertea GM, Antonescu CM et al. StringTie enables improved reconstruction of a transcriptome from RNA-seq reads. *Nat Biotechnol*. 2015;**33**:290-295.

14. Jones P, Binns D, Chang HY et al. InterProScan 5: genome-scale protein function classification. *Bioinformatics*. 2014;**30**:1236-1240.

15. Li H, Handsaker B, Wysoker A et al. The Sequence Alignment/Map format and SAMtools. *Bioinformatics*. 2009;**25**.

16. Goel M, Sun H, Jiao WB et al. SyRI: finding genomic rearrangements and local sequence differences from whole-genome assemblies. *Genome Biol*. 2019;**20**:277.

17. Marçais G, Delcher AL, Phillippy AM et al. MUMmer4: A fast and versatile genome alignment system. Darling AE, ed. *PLOS Comput Biol*. 2018;**14**:e1005944.

18. Emms DM, Kelly S. OrthoFinder: phylogenetic orthology inference for comparative genomics. *Genome Biol*. 2019;**20**:238.

19. McKenna A, Hanna M, Banks E et al. The Genome Analysis Toolkit: a MapReduce framework for analyzing next-generation DNA sequencing data. *Genome Res*. 2010;**20**.

20. Yang R, Guo X, Zhu D et al. Accelerated deciphering of the genetic architecture of agricultural economic traits in pigs using a low-coverage whole-genome sequencing strategy. *GigaScience*. 2021;**10**:giab048.

21. Bu L, Wang Q, Gu W et al. Improving read alignment through the generation of alternative reference via iterative strategy. *Sci Rep*. 2020;**10**:18712.

22. Danecek P, Auton A, Abecasis G et al. The variant call format and VCFtools. *Bioinformatics*. 2011;**27**:2156.

23. Chang CC, Chow CC, Tellier LC et al. Second-generation PLINK: rising to the challenge of larger and richer datasets. *GigaScience*. 2015;**4**:7.

24. Price MN, Dehal PS, Arkin AP. FastTree 2 – approximately maximum-likelihood trees for large alignments. *PLoS ONE*. 2010;**5**:e9490.

25. Alexander DH, Lange K. Enhancements to the ADMIXTURE algorithm for individual ancestry estimation. *BMC Bioinformatics*. 2011;**12**:246.

26. Zhou Y, Zhang Z, Bao Z et al. Graph pangenome captures missing heritability and empowers tomato breeding. *Nature*. Published online June 8, 2022:1-8.

27. Pickrell JK, Pritchard JK. Inference of population splits and mixtures from genome-wide allele frequency data. Tang H, ed. *PLoS Genet*. 2012;**8**:e1002967.

28. Pavlidis P, Živković D, Stamatakis A et al. SweeD: Likelihood-Based Detection of Selective Sweeps in Thousands of Genomes. *Mol Biol Evol*. 2013;**30**:2224-2234.
